# H3K4me3 Histone ChIP-Seq Analysis Reveals Molecular Mechanisms Responsible for Neutrophil Dysfunction in HIV-Infected Individuals

**DOI:** 10.3389/fimmu.2021.682094

**Published:** 2021-07-15

**Authors:** Paweł Piatek, Maciej Tarkowski, Magdalena Namiecinska, Agostino Riva, Marek Wieczorek, Sylwia Michlewska, Justyna Dulska, Małgorzata Domowicz, Małgorzata Kulińska-Michalska, Natalia Lewkowicz, Przemysław Lewkowicz

**Affiliations:** ^1^ Department of Neurology, Laboratory of Neuroimmunology, Medical University of Lodz, Lodz, Poland; ^2^ Department of Biomedical and Clinical Sciences, ‘Luigi Sacco’, University of Milan, Milan, Italy; ^3^ Department of Neurobiology, Faculty of Biology and Environmental Protection, University of Lodz, Lodz, Poland; ^4^ Laboratory of Microscopic Imaging and Specialized Biological Techniques, Faculty of Biology and Environmental Protection, University of Lodz, Lodz, Poland; ^5^ Genomed SA, Warsaw, Poland; ^6^ Department of Periodontology and Oral Mucosal Diseases, Medical University of Lodz, Lodz, Poland

**Keywords:** neutrophils, human immunodeficiency virus, H3K4me3, ChIPSeq, innate immunity

## Abstract

Peripheral neutrophils in HIV-infected individuals are characterized by impairment of chemotaxis, phagocytosis, bactericidal activity, and oxidative burst ability regardless of whether patients are receiving antiretroviral therapy or not. Neutrophil dysfunction leads not only to increased susceptibility to opportunistic infections but also to tissue damage through the release of reactive oxygen species (ROS), proteases, and other potentially harmful effector molecules contributing to AIDS progression. In this study, we demonstrated high levels of histone H3 lysine K4 trimethylated (H3K4me3) and dysregulation of DNA transcription in circulating neutrophils of HIV-infected subjects. This dysregulation was accompanied by a deficient response of neutrophils to LPS, impaired cytokine/chemokine/growth factor synthesis, and increased apoptosis. Chromatin immunoprecipitation sequencing (ChIPseq) H3K4me3 histone analysis revealed that the most spectacular abnormalities were observed in the exons, introns, and promoter-TSS regions. Bioinformatic analysis of Gene Ontology, including biological processes, molecular function, and cellular components, demonstrated that the main changes were related to the genes responsible for cell activation, cytokine production, adhesive molecule expression, histone remodeling *via* upregulation of methyltransferase process, and downregulation of NF-κB transcription factor in canonical pathways. Abnormalities within H3K4me3 implicated LPS-mediated NF-κB canonical activation pathway that was a result of low amounts of κB DNA sites within histone H3K4me3, low NF-κB (p65 RelA) and TLR4 mRNA expression, and reduced free NF-κB (p65 RelA) accumulation in the nucleus. Genome-wide survey of H3K4me3 provided evidence that chromatin modifications lead to an impairment within the canonical NF-κB cell activation pathway causing the neutrophil dysfunction observed in HIV-infected individuals.

**Graphical Abstract:**
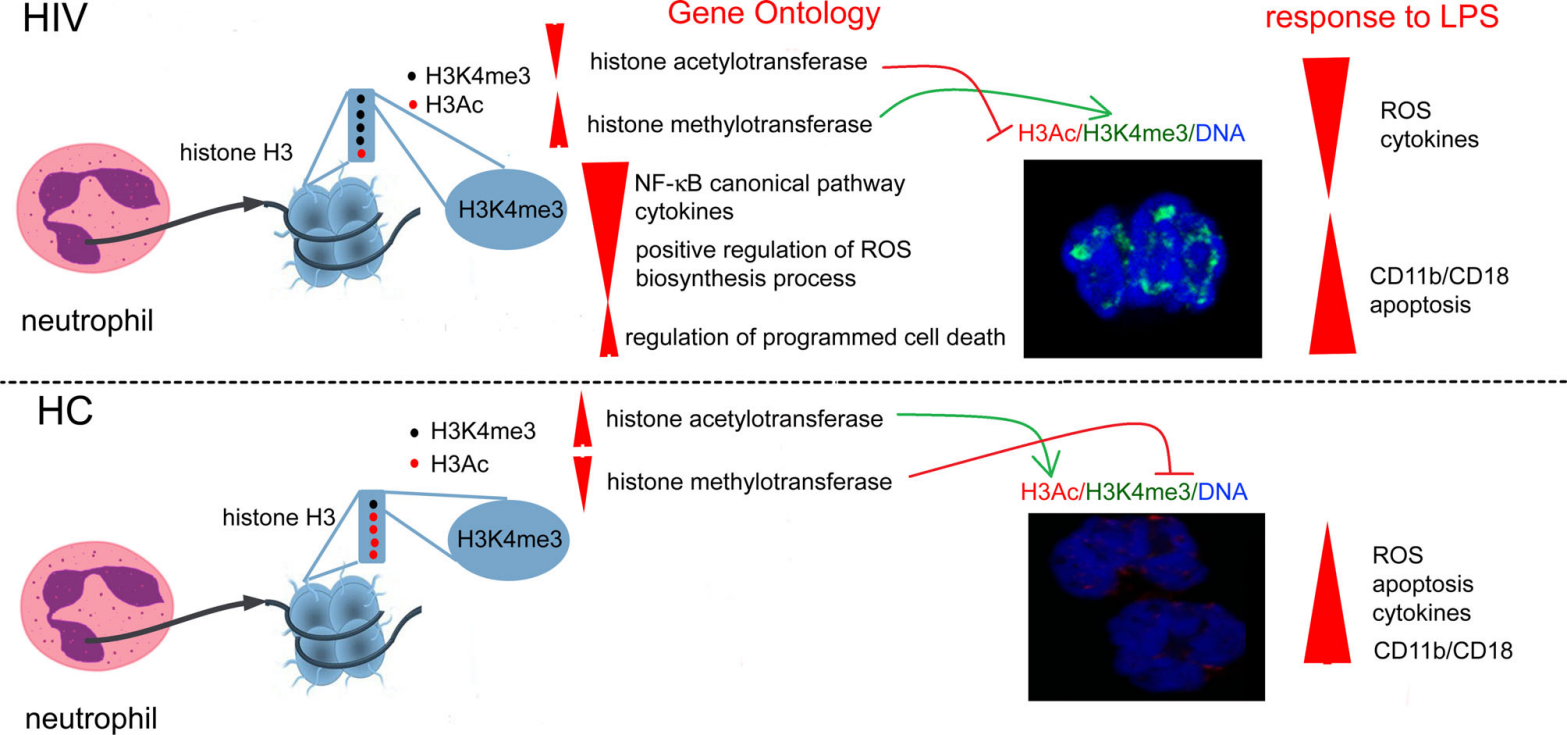
Overexpression of histone H3K4me3-marked in HIV individuals leads to impaired regulation of main cellular processes critical for obtaining high antimicrobial activity by neutrophils. Due to the fact that the posttranscriptional modifications of H3K4me3 histone are also activated via histone H3K4me3-marked itself, this process is enhanced in a positive feedback loop manner and is probably irreversible.

## Introduction

Neutrophils, the most abundant peripheral blood cells of the immune system, are the first responders to most infections (1). In response to pathogens, neutrophils migrate from the blood to the site of microbial invasion, where their activation drives microbicidal mechanisms such as the release of proteolytic enzymes, antimicrobial peptides, and rapid production of reactive oxygen species (ROS). Although neutrophils are not the direct target of HIV, they can contribute to HIV infection pathomechanism in diverse ways. Before the infection, the presence of neutrophil-associated proteins and cytokines in genital tissues was found to associate with HIV acquisition (2). During HIV infection, these cells may, in an uncontrolled way, release proinflammatory mediators in response to the gut bacteria (3), a phenomenon frequently encountered in HIV patients. In such conditions, neutrophil prompt reactions can contribute to the permanent inflammation observed even in patients with undetectable viral load during antiretroviral therapy (ART) or without it (4), a phenomenon known as microbial translocation. On the contrary, some studies demonstrated that neutrophil involvement in HIV infection pathogenesis was mainly associated with their low frequencies and proapoptotic state (5), reduced antimicrobial activity (6), and contribution to the immunosuppression by enhanced release of amino acid depleting enzymes (7) and PD-L1 mediated suppression of T cells (8). Enhanced immunosuppressive activity of neutrophils in already immune deficient conditions together with reduced antimicrobial function was related to higher risk of the secondary infections in HIV positive patients (9). The basis of the dual function of the neutrophils during HIV infection have not been studied and, besides potential differences between peripheral blood and tissue neutrophils (9, 10), it can be a result of the specific molecular pattern of two main groups of determinants: the transcription factors with the central role of NF-κB in induction of a wide spectrum of proinflammatory genes and the presence of cell type-specific patterns of genes associated with chromatin organization (11).

The DNA is folded into nucleosomes comprising approximately 147 bp of DNA and wrapped around a histone octamer. Specific chromatin configuration enables diversified access and activity of regulatory elements, which determines unique cellular phenotypes and guarantees the plasticity of immune cells to adequately respond to external factors (12–14). Neutrophil nucleus is characterized by loosely arranged chromatins, which not only allows for faster formation of neutrophil extracellular trap (NET) cross-links at the site of inflammation, but also easier access of transcription factors to DNA. Although the high plasticity of the nucleus allows neutrophils to react rapidly to invading pathogens, during chronic inflammation, this property may be undesirable, resulting in nonspecific activation. Therefore, analysis of the reorganization of a ‘chromatin landscape’, especially transcriptional start sites (TSSs) of inflammatory genes associated with the activation of neutrophils, may provide a critical advance in understanding HIV-related malfunction of neutrophils. In humans, a strong positive correlation between H3K4me3 and H3K27ac modification of chromatin in relation to activation of immune cells in response to different stimuli was revealed (15, 16). Enhancers, which are classically defined as cis-acting DNA sequences that can increase gene transcription, are typically located far from TSSs and are characterized by the presence of specific post-translational modifications of H3K27ac histones. Contrary to enhancers, TSSs are located mainly in H3K4me3 domains (17). Rapidly activated genes are connected with constitutively high histone H3 acetylation, while genes with slower recruitment of NF-κB and slower kinetics are characterized by low to undetectable baseline level of H3 acetylation. This phenomenon may be responsible for physiologic pre-activation of leukocytes determining stronger and more rapid reaction to pathogens during inflammation. Pre-activation, which is well-described in macrophages, is also observed in circulating neutrophils during inflammation. Macrophages preactivated with IFN-γ before LPS stimulation were found to increase acetylation of ‘slow genes’ and switch them into fast NF-κB recruitment (18). In chronic inflammation, inadequate activation of immune cells can lead to the persistent activation of H3K4me3 domains resulting in disease progression (19, 20).

In this study, we used chromatin immunoprecipitation sequencing (ChIP-Seq) analysis of H3K4me3-marked histone to identify various TSSs associated with NF-κB-mediated response in peripheral blood neutrophils in HIV-infected individuals. We provide comprehensive epigenome analysis of histone H3K4me3 modification in HIV individuals and healthy controls. Observed differences allowed us to identify DNA regions corresponding to pathological activation of neutrophils in HIV-infected individuals. Specific differences within H3K4me3-marked histone corresponded with NF-κB-dependent gene expression as well as biological processes corresponded with their target genes, leading together to the decreased antimicrobial properties of neutrophils and proinflammatory cytokine synthesis driving inflammation.

## Materials and Methods

### Patients and Samples

Fourteen HIV-infected individuals (two females and 12 males; median age 39.9) were diagnosed and recruited at the ‘Luigi Sacco’ hospital, University of Milan. All HIV-infected individuals were naïve to highly active antiretroviral therapy (HAART). Median Log values of viral load in these patients was 4.11 copies/uL, ranging 2.4-6.3 copies/uL, CD4^+^T cells absolute counts ranged from 219 to 1115/µL, with median 514 cells/uL, and CD4/CD8 median value was 0.48 with a range from 0.11 to 1.35. Twelve age- and gender-matched healthy controls (HC) were recruited.

The appropriate Institutional Ethics Committee approved all protocols and informed written consent was obtained from all participants: HIV-infected individuals (approval number 433/08/25/AP, Comitato Etico Locale ET/nb, Ospedale Luigi Sacco, University of Milan) and healthy volunteers (approval number RNN/25/15/KE, Medical University of Lodz).

### Neutrophil Isolation

20 mL of the whole blood on lithium heparin anticoagulant was collected from HIV-infected individuals and HC. Neutrophils were purified by negative selection by microbeads, which allowed the removal of DCs, B cells, monocytes, macrophages, activated T cells, and activated NK cells (MACSxpress Whole Blood Neutrophil Isolation Kit; cat. 130-104-434). Residual erythrocytes were lysed with the use of 2mL ammonium chloride Lysing Reagent (BD cat. 555899) for 5 minutes. The final purity of PMN population was assessed by flow cytometry using CD14-PE (clone M5E2), CD15-FITC (MMA), and CD16-PECy7 (3G8, all from BD Pharmingen) mAbs. Flow cytometric analysis of isolated populations of cells showed that the percentage of CD15^high^CD16^+^CD14^-^ neutrophils was >98%. The level of contaminating CD14^+^CD15^+^ monocytes was about 0.4% and CD15^+^CD16^-^ eosinophils was <0.1% after isolation (**Figure S1A** in **Supplementary Material**). 2x10^6^ neutrophils were incubated without stimulation, in the presence of 100ng/mL ultrapure LPS from *E. coli* (serotype R515, Alexis Biochemicals) in RPMI 1640 for 6 h (5%CO_2_, 37°C, humid atmosphere). For DNA isolation, samples of isolated neutrophils were frozen and kept at -150°C.

### Reactive Oxygen Species (ROS) Production

To avoid isolation-dependent activation of neutrophils, ROS production was assessed by means of the whole blood luminol-enhanced chemiluminescence (CL) using MLX Microtiter Plate Luminometr (DYNEX, USA). The experiments were performed in the non- and LPS-stimulated (10 ng/mL for 10 min in RT before analysis) neutrophils (21).

The results were expressed as Relative Light Units (RLU) corrected by the whole blood neutrophil amounts and haemoglobin concentration according to the formula:

$$CL\;calculated=CL\;measured\;[RLU\;\max]\times \frac{Hb[\%]}{WBC[10^{3}/100\mu L]\times \text{PMN}[\%]}$$

WBC – white blood cell

CL – chemiluminescence

Hb – haemoglobin

PMN – polymorphonuclear leukocytes

### CD11b/CD18 Expression

200 µL isolated neutrophils (2x10^6^ cells/mL in PBS) were incubated at RT with conjugated monoclonal antibodies: anti-CD11b-PE (ICRF44, BD) and CD18-FITC (L130, BD). After 30 minutes of incubation and rinsing, the samples were fixed with 1% paraformaldehyde and analyzed (LSRII, BD).

### Immunocytochemical Analysis (ICC)

For ICC analysis, neutrophils were transferred to gelatin-coated microscope slides by cytospin (300xg, 10 min) and fixed with 4% formaldehyde solution for 20 min at RT. Fixed cells were washed with PBS and blocked with 10% rabbit blocking serum (Santa Cruz Biotechnology, Dallas, TX, USA) supplemented with 3% TritonTM X-100 (Sigma-Aldrich, St. Louis, MO, USA) for 45 min at RT. Next, they were washed and double stained for NF-κB/IκB, H3K4me3/NF-κB, AnnexinV/Caspase3, or H3K4me3/H3Ac. Anti-H3K4me3 (2 µg/mL, clone CMA304, mouse, cat 05-1339 Millipore, Temecula, USA), Anti-H3Ac (Lys4, rabbit, cat 08-539 Millipore, Temecula, CA, USA), anti-NFκB p65 (RelA) (1:100, C-20, rabbit, cat. sc-372, Santa Cruz Biotechnology, Dallas, TX, USA), anti-NFκB p65 (RelA) (1:100, F6, mouse, cat. sc-8008, Santa Cruz Biotechnology, Dallas, TX, USA), anti-IκB (1:100, H4, mouse, cat. sc-1643, Santa Cruz Biotechnology, Dallas, TX, USA), Annexin V (1:100, H-3, mouse, cat. sc-74438, Santa Cruz Biotechnology, Dallas, TX, USA), anti-Caspase 3 (2 µg/mL, 9H19L2, rabbit, cat. 700182, Invitrogen, USA), and rat IgG2b kappa (eB149/10H5 eBioscience) as negative isotype control, were used. All antibodies were suspended in PBS supplemented with 1.5% blocking rabbit serum and 0.3% Triton X-100, 0.01% sodium azide, and incubated overnight at 4°C. Cells were washed and secondary fluorescent Abs were added for 1h at RT: goat pAb to mouse TR (5 µg/mL, cat. T862, Invitrogen, USA) with goat pAbs to rabbit FITC (2 µg/mL, cat. F2765, Invitrogen, USA) or goat pAb to mouse FITC (1:100, cat. ab97239, Abcam) with goat pAbs to rabbit TR (4 μg/mL, cat. T-2767, Invitrogen, USA). For nuclei DNA staining, DAPI (1.5 µg/mL UltraCruz Mounting Medium, Santa Cruz Biotechnology, Dallas, TX, USA) was used. The confocal laser scanning microscopy platform TCS SP8 (Leica Microsystems, Germany) with the objective 63×/1.40 (HC PL APO CS2, Leica Microsystems, Germany) was used for microscopic imaging. Leica Application Suite X (LAS X, Leica Microsystems, Germany) was used for cell imaging. Fluorescence intensity was determined as the arbitrary units (a.u.) of the sum of the fluorescence from all segments divided by the number of segments. The average fluorescence was calculated using at least 100 single cells for each sample. The level of baseline fluorescence was established individually for each experiment. Nonspecific fluorescence (signal noise) was electronically diminished to the level where nonspecific signal was undetectable (22). ICC data were additionally presented as the values of overlap coefficient that indicates the overlap of the fluorescence signals between the channels FITC, TR, and DAPI (nucleus). It was calculated as the mean value from every single Region of Interest (ROI) using Leica Microsystem (LAS - X, ver. 3.7.020979 software, Leica, Germany). The overlap coefficient ranges from 0 (no co-localization) to 1 (complete co-localization).

### Chromatin Immunoprecipitation (ChIP)

Cells were cultured in T75 Nunc flasks in RPMI medium for 8h. ChIP was carried out in neutrophils according to the manual for Magna ChIP™ A/G Chromatin Immunoprecipitation Kit (Merck Millipore, cat.17-1010). Cells were fixed with 1% formaldehyde in RPMI solution for 10 min. at RT, which was quenched with 10x glycine in 5-minute incubation at RT to stop the fixation. After washing with cold PBS, cells were treated sequentially with 1x Protease Inhibitor Cocktail II, Lysis Buffer with Protease Inhibitor Cocktail II, and Protease Inhibitor Cocktail II with Nuclear Lysis Buffer. Next, supernatant was carefully removed and the cell pellet was resuspended in Nuclear Lysis Buffer. Sonication (10 cycles; 30sec. “ON” 30sec. “OFF”) was done using Bioruptor^®^ Pico Sonicator (Diagenode, Belgium). The obtained chromatin was spun at a minimum of 10,000 x g at 4°C for 10 minutes to remove insoluble material. Each immunoprecipitation required the addition of Dilution Buffer and Protease Inhibitor Cocktail II. 25 µL of the diluted chromatin as ‘Input’ was saved at 4°C for further proceeding. Chromatin immunoprecipitation was performed with the use of a set of antibodies: Normal mouse IgG (negative control), anti-RNA Polymerase II (clone CTD4H8) as positive control, and anti-trimethyl-Histone H3 (Lys4) (MC315, Merck Millipore, cat. 04-745) mAbs. Both antibodies were recommended for the use in ChIP-Seq technique (23). Immunoprecipitation reactions were incubated overnight at 4°C with rotation. DNA was eluted with the use of ChIP Elution Buffer/RNase A mixture and purified using spin columns. The DNA concentrations of obtained samples were measured by Qubit 4 Fluorometer (ThermoFisher Scientific).

### Library Preparation and NGS Sequencing

Double-stranded DNA was generated from a single-stranded fraction of ChIPed DNA using NEBNext^®^ Ultra™ II Non-Directional RNA Second Strand Synthesis Module (E6111S,New England Biolabs). Reaction was carried out in the presence of random primers from NEBNext^®^ RNA First Strand Synthesis Module (E7525, New England Biolabs). Libraries for sequencing were prepared using NEBNext^®^ Ultra™ II DNA Library Prep Kit for Illumina^®^ (E7645L, New England Biolabs). Single-end sequencing with read length of 75 bases (SE75) was performed with NextSeq550 (Illumina) in order to obtain at least 20 million reads per sample that could be mapped to the human genome (24). ChIPseq library quality control analysis is presented in Supplementary Materials (**Figure S1B**).

### Bioinformatic Methodology of the ChIPseq Analysis

In the first stage, the quality of the raw sequence reads was checked using the FASTQC software (version: 0.11.8). Next, all reads were subjected to the adapter and quality filtering (minimum quality (-q 25), minimum length (-m 15)) using the Cutadapt tool (version: 1.18) in NextSeq reads mode. Trimmed reads were aligned to the reference genome (GRCh38) using the Bowtie2 (version: 2.2.9) in the single-end mode. Duplicated reads were located and tagged using the Picard MarkDuplicates tool (version: 2.18.4). Reads with low mapping quality score (MAPQ <10) were removed from downstream analysis with the Samtools software (version: 1.6). Protein binding sites identification in the previously prepared BAM files was performed with the MACS2 (Model-based Analysis of ChIP-seq) software (version: 2.1.0) in narrow peak mode (25). Subsequently, identified peaks were annotated using annotatePeaks.pl from Homer software (version: 4.11.1, hg38 annotation library). Additionally, a functional enrichment analysis for various categories (e.g. gene function, biological pathways, domain structure, etc.) was executed (26). To find enriched motifs in ChIPseq peaks the findMotifsGenome.pl program from Homer software (version: 4.11.1) was used. The quantitative assessment of ChIPseq quality was checked applying the ChIPQC package (version: 1.21.0) from R Bioconductor (version: 3.6.0) (**Figure S1C** in **Supplementary Material**). Differentially enriched sites between two experimental conditions were identified using the DiffBind package (version: 2.12.0) from R Bioconductor (version: 3.6.0).

### Human Chemokine Multiple Profiling Assays

Chemokine and cytokine concentrations in neutrophil culture supernatants were measured using Bio-Plex Pro™ Human Chemokine Assays (Bio-Rad Laboratories). Standards and samples were diluted (1:4) in sample diluent and transferred to the plate containing magnetic beads for 1h at RT. The plate was washed (3x) and detection antibody was added for 30 min on a shaker (850 rpm) at RT. After that, the plate was washed (3x) and streptavidin-PE solution was added for 10 min. Subsequently, the plate was washed (3x) and samples were re-suspended in 125 µL of assay buffer and analyzed within 15 min. All samples were analyzed at the same time in duplicates. All reagents and technology were provided by Bio-Rad Laboratories (Bio-Plex 200).

### Multiple Gene Profiling Microarray

168 genes’ expression was analyzed using Human NF-κB Signaling Pathway RT2 Profiler PCR Array and NFκB Signaling Targets RT2 Profiler PCR Array (cat. PAHS-025 and PAHS-225, both Qiagen, UK). cDNA was amplified in the presence of specific primers (RefSeq accession numbers provided in **Supplementary Table 5**) and coated in 96-well microtiter plates on a 7500 Real Time PCR System (Applied Biosystems) according to the following program: 95°C, 10 min (activation of HotStart DNA polymerase); 50 cycles of (95°C, 15s; 60°C, 60s). We used RT2 Real-Time™ SYBR Green/PCR Master Mix (Qiagen, UK) that contains all of the reagents and buffers required for qRT-PCR. The mean expression levels of the following housekeeping genes were used for the normalization of the cDNA samples: hypoxanthine phosphoribosyltransferase 1, β-actin, and glyceraldehyde-3-phosphate dehydrogenase. Data from real-time PCR were calculated using the ∆∆Ct method and the PCR Array Data Analysis Template v3.0 (Qiagen, UK).

### Statistics

Arithmetic means and standard deviations were calculated for all parameters. Statistical verification was made using the Kolmogorov-Smirnov normality test and the Fisher’s test. Statistical significance of differences among the groups was determined by the t-Student test and Cochran test (parametric distributions) or the Wald-Wolfowitz runs test and the Wilcoxon’s rank sum test (non-parametric distributions).

## Results

### Peripheral Blood Neutrophils Isolated From HIV-Infected Individuals Are Characterized by Impaired Antimicrobial Functions

**Figure 1:**
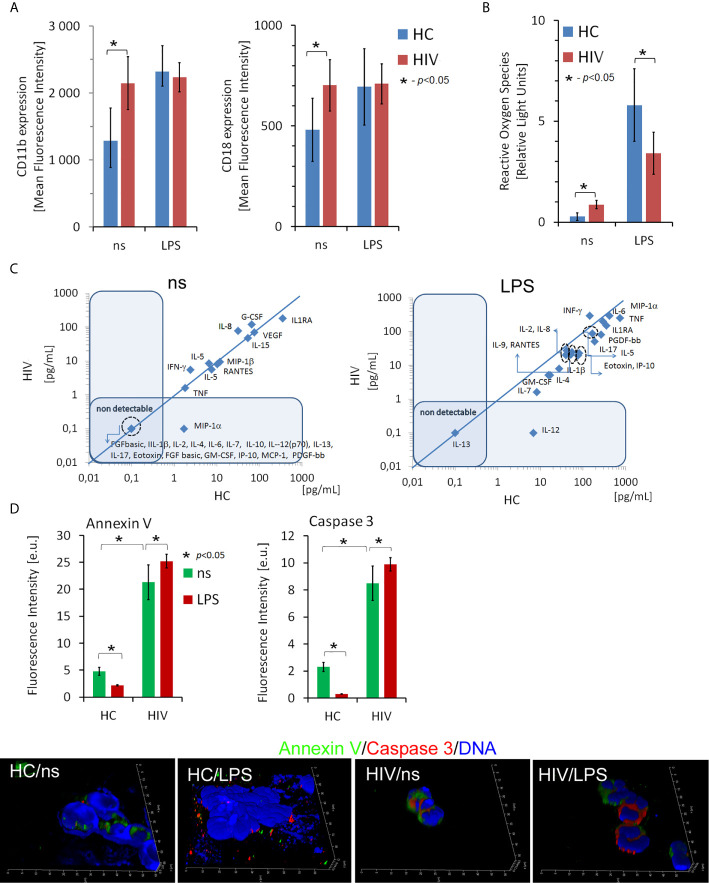
Dysfunction of non-stimulated and LPS-stimulated HIV neutrophils. **(A)** Expression of CD11b and CD18 adhesion molecules on the surface of neutrophils in HIV *vs.* HCs. The bars represent the mean of fluorescence intensity ± SD **(B)** Reactive oxygen species production by non- and LPS-stimulated neutrophils. The bars represent the mean of ROS production in Relative Fold Units ± SD. **(C)** The comparison of the profile of cytokine/chemokine/growth factors released by non-stimulated (left panel) and LPS-stimulated (right panel) neutrophils. The blue line determines the border between up- and down-regulated factors. **(D)** ICC double labeling for caspase-3 (red pseudocolor) and annexin-V (green) revealed that circulating HIV neutrophils are apoptotic. Additional LPS stimulation does not inhibit neutrophil apoptosis as it takes place in HC neutrophils. Nonspecific fluorescence (signal noise) was electronically diminished to the level when nonspecific signal was undetectable (background). The bars represent average fluorescence intensity ± SD calculated from four patients, using at least 100 single cells for each test.

In the initial phase of the study, we analyzed neutrophil effector functions in HIV-infected individuals. The expression of adhesion molecules CD11b and CD18, the ability of neutrophils to generate reactive oxygen species (ROS) and synthetize cytokines/chemokines/growth factors, and the rates of neutrophil apoptosis were analyzed in 8-hour incubations with/without LPS in the HIV individuals and healthy controls (HCs). We showed that the expression of CD11b and CD18 on freshly isolated neutrophils of HIV-infected people was significantly higher than in HCs (**Figure 1A**). Moreover, the expression of these adhesion molecules in HIV-infected individuals but not HCs seemed to be saturated since stimulation of neutrophils with LPS has not caused a further increase in their expression. The analysis of ROS production using the chemiluminescence method revealed that HIV neutrophils were characterized by increased ‘resting’ (non-stimulated) ROS production, while after stimulation with LPS, ROS production ability appeared to be dramatically reduced compared to HC neutrophils (**Figure 1B**). The profile comparison of 27 cytokines/chemokines/growth factors released by unstimulated (**Figure 1C** left panel) and LPS-stimulated neutrophils (**Figure 1C** right panel) revealed significant differences in contrast to HC neutrophils. Unstimulated neutrophils isolated from HIV-infected individuals released significantly higher amounts of IL-8, G-CSF, and IFN-γ and significantly lower amounts of IL-1ra in comparison to HCs. The capacity of LPS-stimulated neutrophils to synthetize cytokines/chemokines/growth factors, with an exception of IFN-γ was dramatically diminished in comparison to HC neutrophils. We detected significantly lower supernatant concentrations of IL-1ra, IL-2, IL-4, IL-5, IL-7, IL-8, IL-9, IL-10, IL-15, IL-17, Eotoxin, FGFbasic, G-CSF, GM-CSF, IP-10, MCP-1, MIP-1α, MIP-1β PDGF-bb, RANTES, TNF, and VEGA (**Table S1** in **Supplementary Material**).

Neutrophils rapidly respond to pathogens but are short-lived cells. One of the effects of their stimulation results in inhibition of apoptosis, an essential process through which neutrophils gain ‘additional time’ necessary for more efficient elimination of pathogens. Using ICC labelling of Annexin V, Caspase 3, and DNA, we demonstrated higher rates of apoptosis in the freshly isolated neutrophils from HIV individuals compared to HCs. Moreover, in contrast to the neutrophils of HCs, LPS stimulation of HIV neutrophils did not inhibit their apoptosis (**Figure 1D**).

To summarise, these experiments demonstrated impaired ability of HIV neutrophils to develop the appropriate reaction following LPS stimulation. Diminished neutrophil function can be an important aspect in increasing the susceptibility of HIV-positive individuals to opportunistic infections. We selected the three samples most accurately representing each group in terms of functional test values (closest to the mean values in HIV+ and HC groups) for further ChIPseq analysis.

### HIV Neutrophil H3K4me3-Marked Histones Are Characterized by Large Fluctuations Within DNA Annotation

**Figure 2:**
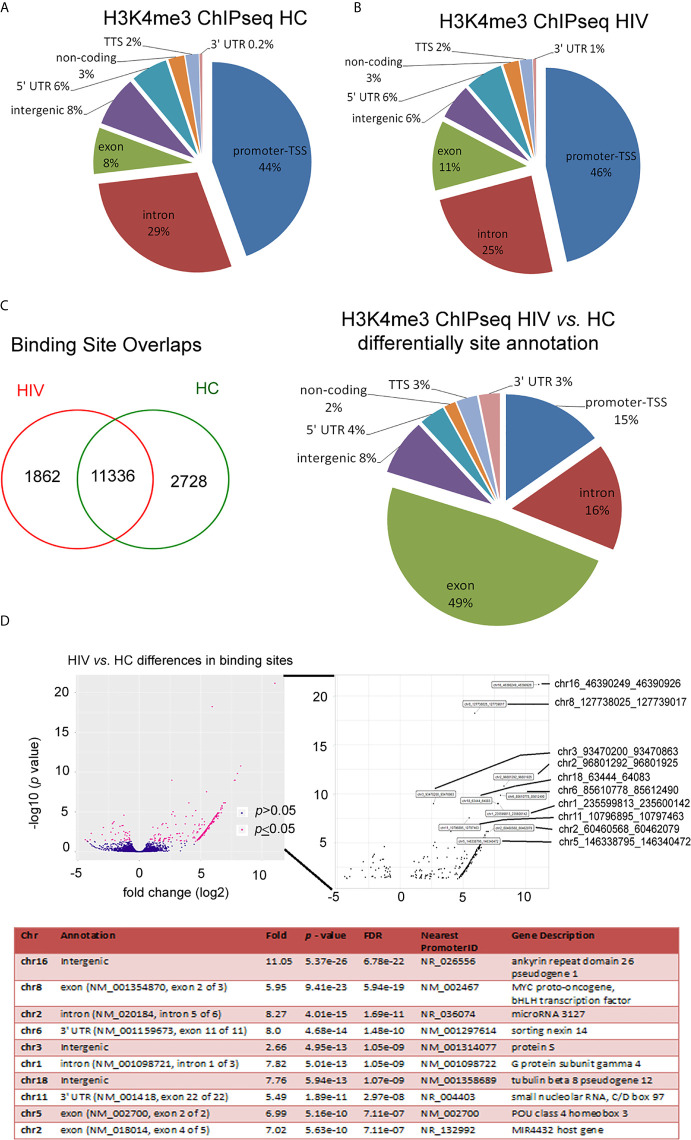
HIV neutrophil H3K4me3-marked histones are characterized by large fluctuations within DNA annotation. **(A)** Pie chart analysis of separate samples within H3K4me3 in HIV and HCs showed only minor differences in DNA annotation of genomic regions. **(B)** Binding sites overlap analysis revealed 1,862 DNA sequences associated with H3K4me3 that specific characterize HIV patients. **(C)** Compression of HIV *vs.* HCs demonstrated the most important changes in exon, intron and promoter TSS genomic features. **(D)** Analysis of statistic differences in protein binding to the H3K4me3 region revealed that most of the binding sites in HIV neutrophils are upregulated. Left and bottom panel show statically significant differences in binding sites as enlarged region. Low panel displays ten peaks with highest statistically significant differences and lowest FDR. More details and all peaks with statistically significant differences are presented in the **Supplementary Table S2**.

A deficient functional response of HIV neutrophils to LPS stimulation suggests some changes in functional genome organization at the stage of posttranscriptional histone modification. The nucleosome H2A, H2B, H3, and H4 histones, extended with other variants, their positioning to each other and chemically modifications defined as ‘nucleosome code’ orchestrate inactive or active transcription (27). Among the over 20 sites of methylation that have been identified on the core histone, the posttranscriptional modification of histone H3 lysine K4 trimethylation is associated with the 5’ open reading frame and directly corresponds to mRNA expression profiling of inflammatory genes. In the next step of our investigation, we focused on the changes within H3K4me3-marked histone in non-stimulated neutrophils using ChipSeq technique. DNA annotation, which describes the function of detected DNA in H3K4me3-marked regions, revealed only slight differences between the HIV and HC groups. Active transcriptional sites (TSSs) were only 2% more prevalent in HIV individuals (**Figure 2A**). Binding sites overlap analysis of allocating genes revealed 1832 peaks within H3K4me3 specific for HIV, 2728 for HC and 11,338 shared by both groups (**Figure 2B**). The accurate description of all identified peaks, with the division of particular Pie chart compartments, was attached in **Supplementary Table 2**. Further DNA annotation analysis of the genes specific for HIV-1 and HC showed that the major differences were observed in exons (49%), introns (16%), and TSS (15%) genomic location (**Figure 2C**). In the next step, we performed a comparison of peak densities which belongs to both groups. Based on the computational algorithm described as Model-based Analysis of ChIP-seq (MACS), we selected 254 peaks with highest density and 42 peaks with lowest density for HIV+ compared to HC group. All selected peaks were characterized by high statistical significance and a very low empirical false discovery rate (FDR) value. **Figure 2D’s** upper panel shows Volcano plot (-log10 *p*-value *vs.* log2 fold expression) of the merged HIV+ and HC peaks. The low panel of **Figure 2D** presents the first ten peaks with the highest *p*-value and the lowest FDR. Based on MACS algorithm, all selected peaks have been assigned to appropriate annotation, closest to the promoter ID, distance to TSSs, as well as gene descriptions (**Table S3** in **Supplementary Material**).

### The Changes Within the Histone H3K4me3 Affect the Main Processes Responsible for Antimicrobial Functions of Neutrophils in HIV-Infected Individuals

Numerous changes in the TSS regions affect the metabolic processes of the cell. Consequently, in the next step of our investigation, we performed Gene Ontology (GO) analysis on the biological process, molecular function, cellular components, and pathway interactions (26). GO components displayed many significant differences in the HIV group in comparison to HC. In particular ‘Metabolic process’, ‘DNA binding’, ‘Activity transcription regulator’, ‘RNA binding’, and ‘Nucleoplasm’ terms were in the top twenty processes with the highest statistically significant differences. The pathway interaction analysis also revealed considerable variation in HIV+ group. The most spectacular, which appeared in ten main processes, were: ‘Signaling events mediated by HDAC Class I’, ‘RAC1 signaling pathway’, and ‘Hedgehog signaling events mediated by Gli proteins’ (**Table S4** in **Supplementary Material**).

**Figure 3:**
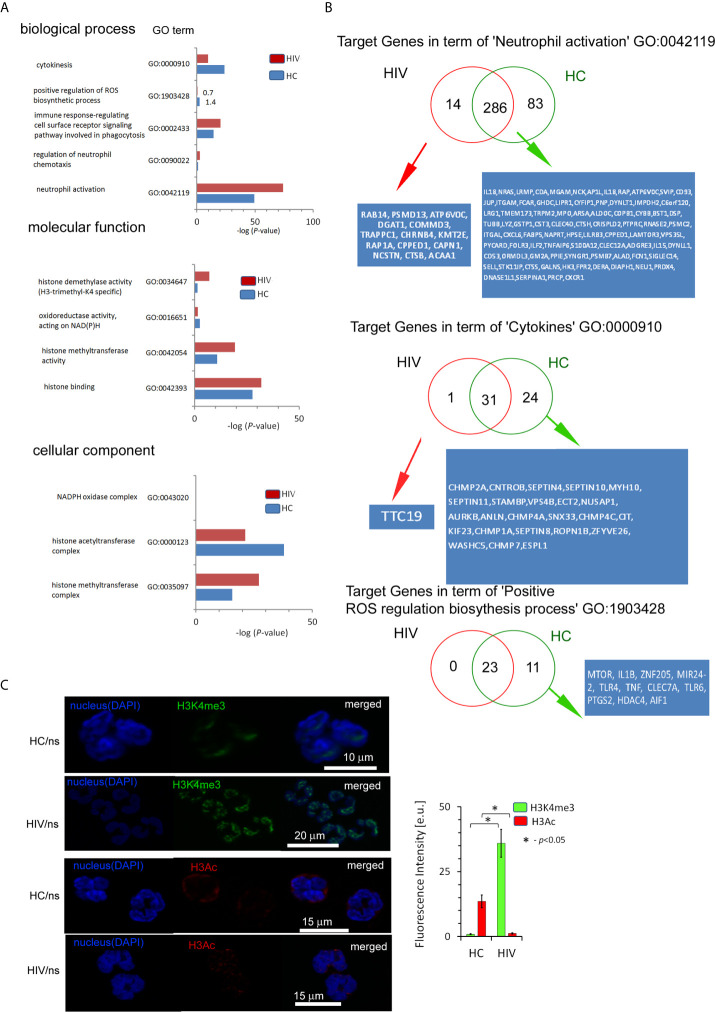
The changes within the histone H3K4me3 affect principle processes responsible for antimicrobial functions of neutrophils in HIV-infected individuals. **(A)** A list of selected biological processes, molecular functions, and cellular components responsible for the ability to neutralize pathogens by neutrophils and in further posttranscriptional histone modifications. The bars represent mean values for HCs (n=3) and HIV-infected individuals (n=3). The analysis of all Gene Ontology (GO) processes with statistic and FDR analysis are provided in the **Supplementary Table S4**. **(B)** The analysis of target genes in the GO term ‘Neutrophil activation’ revealed 14 specific genes in HIV individuals and 83 in HC. The analysis of target genes in the GO term ‘Cytokines’ revealed only specific gene *TTC19* for HIV. **(C)** The ICC analysis with double labeling for H3K4me3 (green pseudocolor) and H3Ac (red) confirmed GO findings suggesting the increased methylation and decreased acetylation process within histone H3 in HIV-infected individuals. Nonspecific fluorescence (signal noise) was electronically diminished to the level when nonspecific signal was undetectable (background). The bars represent average fluorescence intensity ± SD calculated from four patients, using at least 100 single cells for each test.

Next, we searched for the processes directly related to neutrophil activation and histone modification as the primary source of their inflammatory dysfunction. We evidenced that HIV neutrophils were characterized by a reduced amount of target genes within the histone H3K4me3 in terms: ‘Cytokines’, ‘Positive regulation of ROS production’, ‘Oxidoreductase activity acting on NAD(P)H’ and ‘Histone acetyltransferase complex’; and upregulation of target genes in terms: ‘Immune response-regulating cell surface receptor signaling pathway involved in phagocytosis’, ‘Neutrophil activation’, ‘Histone modification’, ‘Histone methyltransferase activity’, and ‘DNA-binding transcription factor activity’. However, no statistically significant differences were demonstrated in ‘NADPH oxidase complex’ (**Figure 3A**). As the major statistical differences were detected in ‘Neutrophil activation’ and ‘Cytokines’, we focused on the particular target genes, and classified them into three groups based on the comparison of peak density. We found 14 specific target genes for HIV neutrophils, 81 for HC, and 281 genes without differences in the process of ‘Neutrophil activation’. In the process of ‘Cytokines’ we noted only one specific target gene (TTC19) in HIV neutrophils, 24 DNA peaks with high density in HC (**Figure 3B**), and 31 without differences. As peripheral neutrophils are characterized by impaired ROS production after LPS stimulation and as we have shown above it is associated with gene disorders within H3K4me3, we focused on the ‘Positive regulation of ROS production’ process. We revealed the deficiency of 11 target genes within H3K4me3-marked in HIV samples including genes coding TLR4 and TLR6 that recognize bacteria and fungi pathogen-associated molecular patterns.

Chromatin organization and regulation of the gene expression allow neutrophils to achieve high plasticity during inflammation. Molecular analyses have demonstrated that this plasticity results from histone posttranslational modifications (HPTMs), mediated predominantly by enzymes catalyzing processes of acetylation and methylation (28). Gene ontology analysis revealed statistically significant histone modification with the predominance of histone H3 methylation over acetylation (**Figure 3A** middle panel). Binding site overlap analysis in the term ‘Histone methyltransferase complex’ revealed 13 DNA peak regions which were particularly characteristic for HIV-infected individuals, 58 DNA peaks as common for both groups, and four DNA peaks present only in HCs (**Table S6** in **Supplementary Material**). In turn, binding site overlap analysis in the term ‘Histone acetyltransferase complex’ revealed one DNA peak region characteristic for HIV-infected individuals, 58 peaks common for both groups, and 18 present only in HCs (**Table S6** in **Supplementary Material**). To validate histone modification, we performed IHC double-staining of non-stimulated neutrophils for H3K4me3 *vs*. H3Ac. We used polyclonal H3Ac antibody that covers the known spectrum of histone H3 acetylation (K4, 9, 14, 18, 23, 27, 36, and 56). This set of experiments revealed high H3K4me3 and low H3Ac fluorescent signals in HIV compared to HC neutrophils, confirming the predominance of H3K4 methylation processes in histone posttranslational modifications (**Figure 3C**).

### Neutrophils of HIV-Infected Individuals Are Characterized by NF-κB Canonical Pathway Disturbances as Well as Decreased Number of NF-κB Binding Sites Within Histone H3K4me3

**Figure 4:**
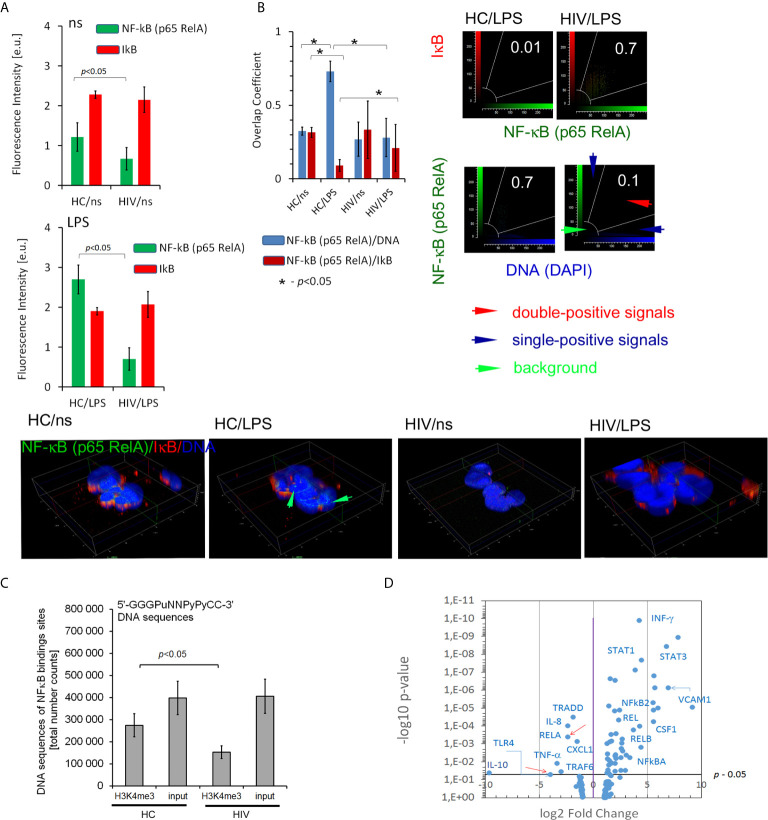
HIV neutrophils are characterized by impaired NF-κB activation dependent on the p65-RelA subunit. **(A)** ICC florescent intensity analysis with double labeling for p65-RelA (green pseudocolor) and IκB (red) in non- and LPS-stimulated neutrophils. The bars represent average fluorescence intensity ± SD calculated from four patients, using at least 100 single cells for each test. (**B** right panel) Overlap coefficient of p65-RelA *vs.* IκB and p65-RelA *vs.* DNA analysis suggests p65-RelA colocalization with its inhibitor IκB which results in disturbance of p65-RelA relocation to the cell nucleus. (**B** Low panel) ICC 3D projection of nucleus visualizes the lack of NF-κB (p65-RelA) colocalization with DNA after LPS-stimulation in HIV individuals. Green arrows show an overlap of signal from NF-κB (p65-RelA) and DNA in HCs. **(C)** Total amount of NF-κB binding sites within histone H3K4me3-marked in non-stimulated neutrophils. The bars represent mean ± SD for HCs (n=3) and HIV-infected individuals (n=3). **(D)** Disturbances in translocation of NF-κB to the nucleus are reflected in the profile of mRNA expression of proinflammatory genes. The mRNA expression of all targets and genes associated with NF-κB are provided in the **Supplementary Table S5**.

High production of ROS by unstimulated neutrophils in HIV individuals and impaired respiratory burst in response to LPS suggests the existence of significant changes of molecular pathways which control these processes. The transcription factor NF-κB plays a critical role in acute inflammation mediated by neutrophils and should be activated only transiently (29). The non-covalent association of IκB (inhibitor of NF-κB) with NF-κB shifts the steady-state subcellular localization of NF-κB dimers to the cytoplasm. Biological inactive NF-κB–IκB complex is interrupted during cell activation by IKK kinase leading to liberation of NF-κB, its translocation to the nucleus, and binding to the site-specific transcription sequences (κB DNA sites). Using IHC double-staining method to detect NF-κB subunit RelA(p65) and IκB, we observed lower expression of NF-κB RelA(p65) in non-stimulated neutrophils of HIV in comparison to HCs. In contrast to neutrophils of HCs, LPS-stimulation of HIV neutrophils did not cause an increase in NF-κB RelA(p65) expression. In both groups, there was no change in IκB expression regardless of whether the cells were stimulated or not (**Figure 4A**). The co-localization analysis of NF-κB RelA(p65) *vs*. IκB and NF-κB RelA(p65) *vs.* DNA after LPS exposure revealed deficiency in the dislocation of NF-κB RelA(p65) to the nucleus as well as a lack of NF-κB RelA(p65) detachment from its IκB inhibitor (**Figure 4B**). Subsequently, we noted statistically significant low amounts of κB DNA sites within histone H3K4me3-marked in HIV-infected individuals compared to HCs (**Figure 4C**). In the last set of experiments, we considered how these changes affect gene expression related to NF-κB activation of cells and those regulating NF-κB activity. We noted 50 genes up- and seven down-regulated as well as 78 without statistical significance in non-stimulated neutrophils isolated from HIV-infected individuals (**Figure 4D** and **Table S5** in **Supplementary Material**). Among others, we observed low mRNA expression for: NF-κB RelA(p65), IL-8, IL-10, and TNF-α (**Figure 4D**; red arrows point NF-κB RelA(p65) and TLR4). mRNA of IκBα IκBβ and IκBε were without changes in non- stimulated neutrophils compared to HCs (**Table S5** in **Supplementary Material**).

**Figure 5:**
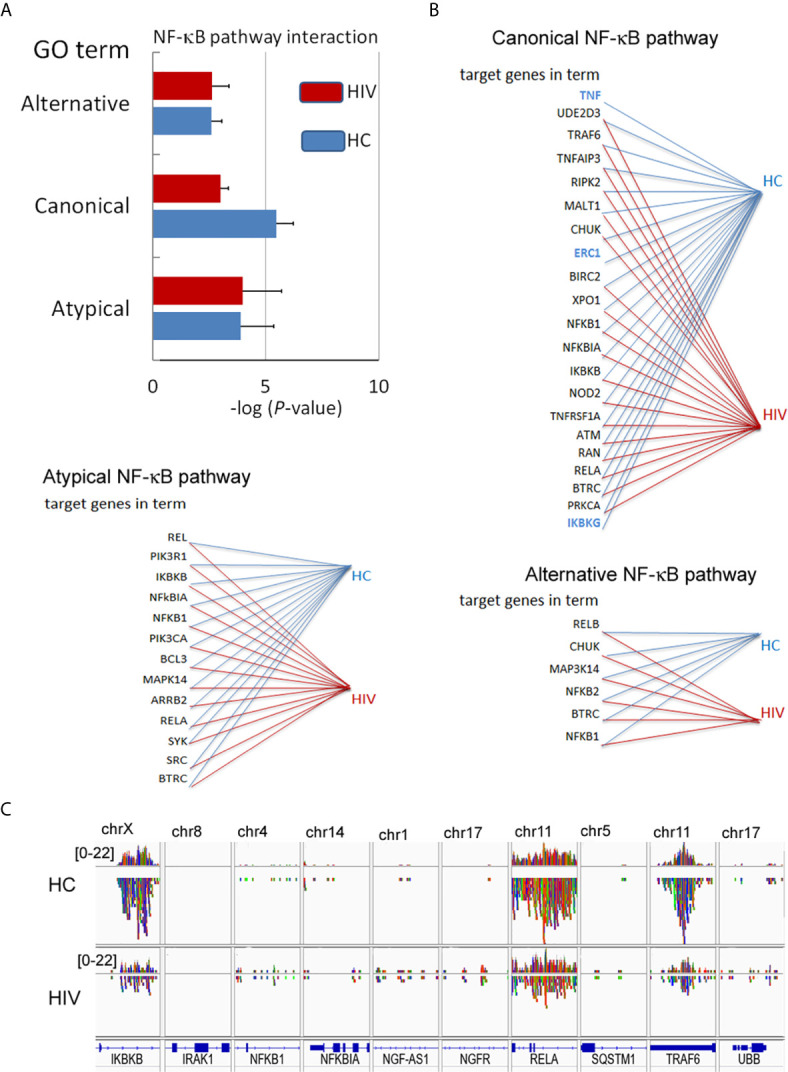
Changes in the DNA within H3K4me3-marked histone affect canonical NF-κB pathways. **(A)** The analyses of GO terms: alternative, canonical, and atypical NF-κB pathways. The bars represent mean values for HCs (n=3) and HIV-infected individuals (n=3) **(B)** The analysis of target genes in the GO term ‘Canonical NF-κB pathway’ indicated the lack of signals for *TNF*, *ERC1*, and *IKBKG* within H3K4me3 in HIV neutrophils (blue color). **(C)** The comparison of selected genes related to NF-κB shows a statistically significant smaller number of NGS readings (low density) within *RelA*, *IKBKB*, and *TRAF6* coding genes related to the GO term ‘Canonical NF-κB pathway’.

In the next step, we verified whether the NF-κB RelA(p65) down-regulation is a result of H3K4me3-marked histone dysregulation. Gene Ontology analysis revealed statistically significant down-regulation of target genes associated with ‘Canonical NF-κB pathways’ term (**Figure 5A**). The canonical NF-κB pathway is stimulated by ligands of diverse immune receptors and involves three processes: IκB kinase (IKK) activation; IκBα phosphorylation; and its ubiquitination and nuclear translocation of NF-κB units such as p50, RelA(p65), and cREL. Detailed analysis of individual target genes associated with this term indicated an undetectable DNA peak in TNF-α, IκBKG, and ERC1 regions in histone H3K4me3-marked (**Figure 5B**). Overlap peak density comparison revealed additional changes within peaks assigned to RelA(p65), TRAF6, and IkBKB genes (**Figure 5C**). In our studies, we also took into consideration other alternative NF-κB pathways. ‘Non-canonical (alternative) NF-κB pathway’ is associated with pre-activation of immune cells in response to signals from a subset of tumor necrosis factor receptor (TNFR) superfamily members and involves slow and persistent activation of NF-κB-inducing kinase (NIK). NIK-mediated p100 phosphorylation results in nuclear translocation of p52 and RelB (30). In turn, ‘atypical NF-κB signaling pathway’ is initiated by genotoxic stress, which in the first step leads to a translocation of NEMO (NF-κB Essential Modulator, IKKγ) to the nucleus where it is sumoylated and subsequently ubiquitinated. This process is mediated by the ataxia telangiectasia mutated kinase, which, in cooperation with NEMO, causes an activation of IKKβ and subsequently NF-κB (31). We did not observe any changes within GO term ‘Atypical NF-κB pathway’ or ‘Alternative NF-κB pathway’ (**Figures 5A, B**). This set of experiments directed on ‘Canonical NF-κB pathway’ as a central point of neutrophil dysfunction in response to LPS suggests that this phenomenon is caused by the changes within H3K4me3-marked histone.

One of the most important processes in binding transcription factors with a chromatin template is destabilization of nucleosomes by SWI/SNF Complex throughout ATP-driven translocation of the protein along nucleosomal DNA (32, 33). In our experiment, we noted statistically important changes within H3K4me3-marked in term ‘SWI/SNF superfamily-type complex (GO:0070603)’ in HIV non-stimulated neutrophils (–20.4 log10 *p*-value in HIV and -17.2 in HCs, respectively, **Table S4** in **Supplementary Material**).

### Changes Within H3K4me3-Marked Histone in HIV Neutrophils Involve Deregulation of Histone 1 Which Is Responsible for DNA Fragmentation Pathway but Not Bcl-2 Pathway

**Figure 6:**
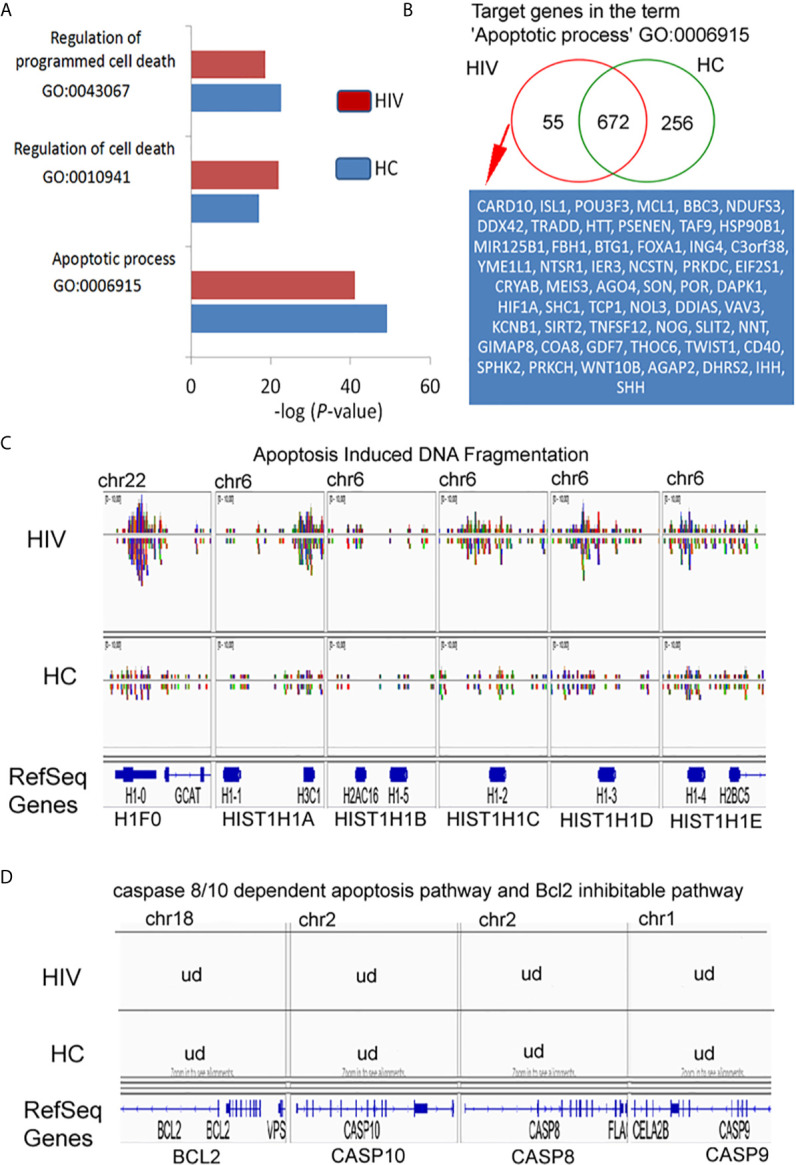
H3K4me3-associated genes affect apoptosis by deregulation of DNA fragmentation but not BCL2- or caspase-regulated execute phase of apoptosis. **(A)** Gene Ontology analysis of apoptosis and cell death. The bars represent mean values for HCs (n=3) and HIV-infected individuals (n=3). **(B)** Binding site overlap in the GO term ‘Apoptotic process’. A detailed list of all target genes is attached in the **Supplementary Table 6**. **(C)** The comparison of peak density in the genes responsible for histone-1-induced DNA fragmentation revealed high density DNA within histone H1 in HIV neutrophils. **(D)** The comparison of peak in the representative genes responsible for execution phase of apoptosis as well as Bcl2 pathway revealed undetectable DNA within H3K4me3. ud- undetectable level of DNA.

In our preliminary experiments, we found that neutrophils of HIV-infected individuals succumb easily to apoptosis. Thus, in the last step of our investigation, we analyzed the Gene Ontology associated with apoptosis. For these analyses were considered neutrophils isolated from HIV-infected individuals with significantly down-regulated expression of genes found within Gene Ontology terms ‘Regulation of programmed cell death’ and ‘Apoptotic process’ (**Figure 6A**). Binding sites overlap in term of ‘Apoptotic process’ analysis revealed 55 DNA peak regions which are particularly characteristic for HIV-infected individuals, 672 as common for both groups, and 256 present only in HC (**Figure 6B**, **Table S6** in **Supplementary Material**). Compression of read density overlap of peak regions highlighted high concentration regions within histone H1 protein’s (H1F0, HIST1H1A, HIST1H1B, HIST1H1C, HIST1H1D, and HIST1H1E) mainly presented on chromosome 6 in the HIV group (**Figure 6C**). We performed a similar comparison for target genes responsible for caspase 8/10- dependent apoptosis and Bcl-2 pathway. We found no differences within investigated regions (**Figure 6D**). These data hypothesized that H3K4me3-marked histone also implies a participation in apoptosis by triggering of H1histone genes’.

## Discussion

Regulation of gene expression depends on histone posttranslational modifications (HPTMs), DNA methylation, histone variants, remodeling enzymes, and effector proteins that influence the structure and function of chromatin. All these processes are interrelated and dependent on each other, creating a specific ‘histone code’ (34) allowing immune cells to achieve high plasticity during inflammation. One of the well-known mechanisms of chromatin remodeling in response to the pathogen relates to the polarization of naive T lymphocytes to CD4 or CD8 subpopulation described by Harrison group (35). Human activated lymphocytes are characterized by coordinated changes at different levels of chromatin architecture. These changes affect all levels of chromatin organization, starting from the primary level of organization of nucleosome-mediated by HPTMs, then, intermediated level where the genome is organized into protein-mediated loops that facilitates interaction between the pairs of promotors and enhancers, and finally, at a high level with the genome organized into self-interacting chromatin (35). Euchromatin of immune cells is characterized by high levels of acetylation and H3K4me1/2/3 (28). It seems highly probable that the mutual proportions of HPTMs as well as the time of particular modifications determine the functional state of different types of leukocytes according to the role played at a specific phase of inflammation. In this study, we have demonstrated in two independent sets of experiments (Gene Ontology of histone H3K4me3-marked annotation analysis and H3K4me3/H3Ac protein expressions) that HIV neutrophils are characterized by increased expression of histone modification process associated with methylation and a simultaneous inhibition of the acetylation. Gene Ontology of H3K4me3-marked histone has revealed overexpression in ‘Histone methyltransferase activity’ term, suggesting that actively transcribed genes are responsible for H3K4me3 modification themselves. As this observation was also confirmed by the high expression of H3K4me3 in ICC method, it seems highly probable that H3K4me3 modification and its intensity are orchestrated by positive feedback reaction. In addition, Gene Ontology of histone H3K4me3 has revealed the downregulation in ‘Histone actetyltransferase activity’ term, which corresponds with low expression of H3Ac protein. As both processes are interrelated and controlled by H3K4me3 in a positive feedback manner, the changes associated with H3K4me3 modification may be definitive and irreversible, determining neutrophil fate during its short lifespan.

Posttranscriptional modification of H3 histone leads to getting access to DNA regions rich in enhancers and promoters. Modification of H3K4me3 is observed at the 5’ open reading frame of actively transcribed genes, while H3 acetylation is localized rather far from specific gene regions other than just at TSSs (20). This is related to physiological mechanisms involved in pathogen eliminations. Rapidly triggered genes have showed constitutively high histone acetylation, while genes recruiting NF-κB with slower kinetics were characterized by low or undetectable basal levels of H3 acetylation. Immune competitive cell pretreatment with low concentrations of pro-inflammatory cytokines (eg. INF-γ and TNF), before LPS stimulation, was found to increase acetylation of slow genes switching them into fast NF-κB pathway (36). We can suspect that a similar phenomenon, described as priming or pre-activation, takes place in neutrophil functioning. The first pro-inflammatory signal of TNF, C5a, or IL-8, applied at a very low concentration for a short period of time, leads to a much more intensive neutrophil ‘burst of ROS’ and phagocytosis in response to pathogens compared with neutrophils without any ‘pre-activation’ (37). Therefore, the low expression of H3Ac-marked histone probably results from dysregulation in neutrophil pre-activation in HIV-infected individuals, causing a deficiency of effective antimicrobial response and low pro-inflammatory cytokine/chemokine/growth factor synthesis after LPS stimulation.

In turn, the increased expression of H3K4me3 causes low-grade permanent gene transcription resulting in enhanced ROS synthesis as well as high expression of CD11b/CD18 adhesive molecules in HIV non-stimulated neutrophils. This observation is also in accordance with Gene Ontology analysis, where significantly increased biological processes ‘Neutrophil activation’, ‘Regulation of neutrophil chemotaxis’, and ‘Immune response-regulating cell surface receptor signaling pathway involved in phagocytosis’ were detected. In addition, an easier access to TSSs is connected with requirement of Swi/Snf chromatin remodeling complex which cooperates with DNA-histone exposing DNA on the nucleosome surface (38, 39). In our study, we have noted high statistical differences within H3K4me3-marked histone in term of ‘SWI/SNF superfamily-type complex (GO:0070603)’ in HIV non-stimulated neutrophils. Under physiological conditions, this process is associated with histone acetylation leading to wide access to TSS regions, while genes with a constitutive nucleosome-accessible configuration in the TSS regions are activated in the absence of SWI/SNF activity (40). Therefore, the high activity of this process observed *in vitro* without H3 acetylation in unstimulated neutrophils of HIV-infected individuals may be the cause of their low-grade permanent activation.

NF-κB affects most aspects of neutrophil cellular physiology associated with an antimicrobial immune response that involves inhibition of neutrophil apoptosis, ROS and proteolytic enzyme production, and neutrophil extracellular trap (NETs) formation responsible for pathogen elimination, and chemokine/cytokine production for recruitment of other immune cells. Five genes encode the entire family of NF-κB transcription factors: NFKB1, NFKB2, RELA, RELB, and REL (41). Their polypeptide products give rise to the mature NF-κB subunits p50, p52, RelA(p65), RelB, and cRel, which combined in pairs produce 15 distinctly functioning NF-κB dimers (42). In this study, we have shown that mRNA and polypeptide RelA (p65) expression are decreased in HIV neutrophils. Other NF-κB mRNA factors were upregulated. This pattern of NF-κB subunits mRNA expression results from the regulation based on a positive feedback manner, as transcription of the genes encoding the NF-kB polypeptides is upregulated during NF-κB activation, with the exception of RelA (43). Next, we addressed the activity of an inhibitor of NF-κB (IκB) that controls NF-κB dimers throughout NFκB-IκB complex. IκBα, IκBβ and IκBε−mediated canonical IκB activities orchestrate NF-kB dimers that contain at least RelA or c-Rel subunits binding in the nucleus. We have shown that IkBα IκBβ and IκBε mRNA expression and polypeptide specific for all subunits of IkB (IkBα IκBβ, IκBε) were without any changes in HIV neutrophils in comparison to HC. To summarise, these data indicate dysregulation in NF-κB activity in HIV neutrophils related to deficiency in RelA subunit but not in IκB expression.

Next, we found that decreased expression of mRNA for RalA in HIV neutrophils corresponded with RelA gene low peak density within histone H3K4me3-marked. The downstream GO analysis of NF-κB function and putative target genes in GO annotation revealed inhibition in the transcription of canonical NF-κB pathway. Detailed analysis of individual target genes has demonstrated the absence of TNF-α, IκBKG, TLR4, TLR6, and ERC1 peaks within H3K4me3-marked histone in HIV *vs*. HC neutrophils. Absence of TLR4 peak within H3K4me3-marked histone in HIV-infected individuals may be the reason for low TLR4 mRNA expression and reduced functional response of HIV neutrophils to LPS.

Further analysis has revealed differences in peak density in HIV neutrophils within RelA, TRAF6 (TNF Receptor Associated Factor), and IκBKB (Inhibitor of nuclear factor kappa B Kinase subunit Beta). These changes within H3K4me3 histone were responsible for both dysfunction of NF-κB–IκB complex and impairment of NF-κB translocation to nucleus. Another aspect that affects activation of genes dependent on the NF-κB activity is the small number of NF-κB binding sites within H3K4me3-marked histone. Dysregulation of NF-κB activity and reduced availability for NF-κB binding sites appear to be two independent phenomena. In our opinion, the reduced number of NF-κB binding sites may arise from the reduction of H3 acetylation processes that control the primary access of NF-κB to promotor/TSS regions (11, 44). Moreover, it was previously demonstrated that the enhancer chromatin signature consists of acetylated histones associated with high levels of H3K4me1 histone modification (45, 46).

Based on this observation, we have concluded that a dichotomy between non- and LPS-stimulated HIV neutrophils is the result of posttranscriptional histone modifications with predominate methylation of H3K4me3 and simultaneous inhibition of acetylation. This may explain why circulating non-activated neutrophils in HIV-infected individuals exhibit some characteristics of activated cells with elevated basic ROS production and high expression of adhesive molecules but, at the same time, are unresponsive to additional stimulation with LPS. Similar observations were made by others showing that isolated neutrophils from untreated HIV patients exhibited increased CD11b and decreased L-selectin (CD62L) expression, increased actin polymerization and ROS production, but a reduced capacity to respond to stimulation (47).

The last aspect of our study was devoted to explanation of abnormal apoptosis observed in circulating HIV neutrophils characterized by the high rate of apoptotic cells and inability of LPS to induce apoptosis inhibition. In the execution phase of apoptosis, effector caspases cleave vital cellular proteins leading to the morphological changes including destruction of the nucleus and other organelles, DNA fragmentation, chromatin condensation, cell shrinkage, cell detachment, and membrane blebbing (48). Our study has demonstrated that neutrophils isolated from HIV patients were apoptotic with high expression of Annexin V as well as caspase-3 in the cytosol and in the nucleus. Surprisingly, this process, in opposition to HC neutrophils, was not only irreversible but even intensified following LPS exposure. As apoptotic neutrophils are characterized by reorganized multidivided nuclei, this process should be associated with a change in chromatin condensation mediated by posttranslational modification of histone H1. Histone H1 protein binds to linker DNA between nucleosomes forming the chromatin fiber which is necessary for the condensation of nucleosome chains (49). In addition, histone H1.2 can translocate to the cytosol and induce apoptosis through a Bak-mediated mitochondrial release of cytochrome C, allowing for caspase activation (50, 51). Gene Ontology analysis of putative genes attendant with executive phase of apoptosis has revealed DNA regions with high peak concentration of histone H1 proteins including H1.2 within H3K4me3 in HIV-infected individuals. Since in H3K4me3-marked histone, no differences in DNA peak concentration of the principal intrinsic pathway components of apoptosis Bcl-2, caspase-8, and -10 have been observed, engagement of H3K4me3 by impelling the histone H1 may point to histone modification of H3K4me3 as a primary process in apoptosis induction. This finding can also explain why neutrophils in HIV-infected individuals were unresponsive to apoptosis inhibition after LPS stimulation. Acceleration of spontaneous neutrophil apoptosis at early stages of untreated HIV patients was also noted by others, displaying the engagement of caspase-3 independently of caspase-8 and suggesting the existence of the intrinsic pathway (52–56).

The limitation of our study is connected with the relatively small group size. We limited our investigation to three selected representatives of the entire group of patients, whose functional neutrophil tests (ROS production, adhesive molecule expression, cytokine production, and apoptosis) were closest to the mean values of all cases taken for our preliminary study. Another limitation of our research is the examination of only one histone in posttranscriptional modification. There are many other possibilities of HPTMs, such as acetylation, methylation, phosphorylation, ubiquitination, SUMOylation, ADP-ribosylation, deamination, and the non-covalent proline isomerization (28), that can also affect the chromatin condensation to organize the genome into transcriptionally active or inactive regions. In HIV neutrophils, histone H3K4me3-marked modified H1 histone regions which may affect chromatin condensation and nucleosome density. Neutrophil nucleus density, in comparison to lymphocytes or NK cells, is characterized by loosely arranged chromatin that facilitate an access of transcription factors to DNA and its remodeling during NETosis. Therefore, neutrophil nucleus density may dynamically change during infection, and ideally, H3K4me3 ChIP-Seq data should be normalized to total H3.

An important aspect which needs to be solved is finding the direct trigger leading to the histone H3 modification. It seems probable that these modifications take place during myelopoiesis. This view is supported by several independent observations. Firstly, there is the large scale of dysregulation in all neutrophil fundamental functions in the relatively short time of their existence in periphery. Secondly, there is the functional homogeneity of neutrophils (relatively low value of standard deviation within average value) within the HIV group. Thirdly, there is the morphological homogeneity observed in FSC *vs.* SSC dot plot of flow cytometry during CD11b/CD18 analysis as well as in ICC performed in freshly isolated neutrophils. Finally, the range of posttranscriptional histone H3 modification observed in HIV neutrophils seems to be too wide to occur just in a few hours of neutrophil existence in the periphery. Potential mechanisms affecting histone H3 modifications during myelopoesis include an altered bone marrow cytokine environment, direct suppression of hematopoiesis by viral proteins, and possible HIV infection of hematopoietic stem cells (57). In this context, activation of interferon-related antiviral immune mechanisms seems to be of particular interest (58, 59). In the animal models of lymphocytic choriomeningitis virus (LCMV) and of poly(I:C) infections, induction of IFN-α/β, but not IFN-γ resulted in reduction of cellularity of hematopoietic progenitors in bone marrow (60). In our study, we found that IFN-α/β synthesis was not elevated in HIV neutrophils, while IFN-γ synthesis was increased not only by LPS- but also non-stimulated cells, suggesting that IFN-γ synthesis may occur at myelopoiesis. Experiments performed by Buro et al. demonstrated that H3K4me3 is increased in parallel with STAT1 (signal transducer and activator of transcription 1) activity after 30’ IFN-γ (5ng/mL) exposition. They also confirm inhibition of global H3K4me3 after IFN-γ stimulation of 2fTGH cells by methyltransferase inhibitor: 5’-deoxy-5’-methyl-thioadenosine (MTA) (61). The effects of IFN on the cell can be also observed through triggered IRF (interferon regulatory factor) molecules (62). We noted high mRNA expression of IRF1, STAT1, as well as INF-γ in non-stimulated neutrophils which indicate possible IFN-γ involvement in the disturbance of HIV neutrophils at the maturating stage of these cells. This hypothesis requires verification.

Another aspect that has not been explored is the participation of histone-modifying enzymes which not only modify the histone proteins, but also play a role in the modification of p65 subunit of NF-κB (63). The six methylated K sites, K37, 218, 221, 310, 314, and 315, of NF-κB (p65, RelA) are modified by different histone-modifying enzymes (63). Verification of these possibilities by analysis of bone marrow cells, an environment affecting myelopoesis as well as histone-modifying enzyme activity during maturation of these cells, could bring us closer to the primary factors responsible for neutrophil dysfunction in HIV-1 individuals.

To summarize, our study has demonstrated that neutrophil dysfunction in HIV-infected individuals is associated with overexpression of H3K4me3 with simultaneous H3Ac downregulation. Overexpression of H3K4me3-marked histone leads to impaired regulation of basic cellular processes critical for obtaining high antimicrobial activity of neutrophils. Due to the fact that the posttranscriptional modifications of H3K4me3 histone are also activated *via* histone H3K4me3-marked itself, this process is enhanced in a positive feedback loop manner and is probably irreversible. Our study allowed us to identify possible target genes within H3K4me3 responsible for the neutrophil dysfunction in HIV-infected individuals, however, the data should be validated on a larger cohort of patients with additional use of standard ChIP-qPCR.

## Data Availability Statement

The ChIPseq data has been deposited to the GEO, accession number GSE169310.

## Ethics Statement

The studies involving human participants were reviewed and approved by (1) HIV-infected individuals (approval number 433/08/25/AP, Comitato Etico Locale ET/nb, Ospedale Luigi Sacco, University of Milan) and (2) healthy volunteers (approval number RNN/25/15/KE, Medical University of Lodz). The patients/participants provided their written informed consent to participate in this study.

## Author Contributions

MT designed, performed, and analyzed experiments (AIDS neutrophil isolation and ROS and adhesive molecules analysis), and carried out manuscript review. PP designed, performed, and analyzed experiments (HC neutrophil isolation, Chromatin Immunoprecipitation, cytokine/chemokine/growth factors profiling assays in both groups, and mRNA concentration analysis). MN performed HC neutrophil isolation, Chromatin Immunoprecipitation, and mRNA concentration analysis (Multiple Gene Profiling Microarray) and contributed to manuscript editing. MD participated in QC DNA shearing analysis by DNA electrophoresis. SM and MW performed ICC analysis. AR enrolled HIV-infected individuals and assisted in manuscript review. MK-M enrolled healthy volunteers. JD performed NGS and bioinformatics analysis. NL prepared the manuscript (review and editing). PL supervised and designed the study and experiments, interpreted the data, and wrote the manuscript (original draft). All authors revised the manuscript. All authors contributed to the article and approved the submitted version.

## Funding

This work was supported by the grants from the National Science Centre 2015/17/B/NZ6/04251.

## Conflict of Interest

The authors declare that the research was conducted in the absence of any commercial or financial relationships that could be construed as a potential conflict of interest.

## Supplementary Material

Supplementary Figure 1
**(A)**. Phenotypic analysis of purified neutrophils used for ChIPseq experiments. Flow cytometry analysis of CD14, CD15 and CD16 in the population of isolated neutrophils. Cells double-positive for CD16 and CD15 were identified as neutrophils, CD14^high^CD15^+^ cells as monocytes and with CD16^-^CD15^+^ cells as eosinophils. **(B)**. ChIPseq library quality control analysis. ChIPseq library was generated from 1 ng ChIP DNA with 18 cycles of PCR. The size of the library distribution is approximately 200-300 bp. **(C)**. ChIPseq quality control report as principal component analysis (PCA). Replicate samples of high quality can be expected to be spatially grouped within the PCA plot. Evaluation of the PCA plot has shown that all samples are suitable and representative for bioinformatic analysis. The analysis was performed by ChIPQC:1.21.0 software (author: Tom Carroll, Wei Liu, Ines de Santiago, Rory Stark).

Supplementary Table 1 The profile of cytokines/chemokines/growth factors released by non- and LPS-stimulated neutrophils in healthy controls and HIV-infected individuals. Data are presented in pg/mL as means ± SD.

Supplementary Table 2 The list of all identified peaks within HIV and HCs divided into HIV specific, HC specific, and common for both groups.

Supplementary Table 3 The list of all statistically significant differences in the peak density between HIV and HCs. The comparison was performed using MACS algorithm. All selected peaks were sorted from the highest statistically significant value to the lowest one. All selected peaks have been assigned to appropriate annotation, closest to the promoter ID, distance to TSSs, as well as gene descriptions.

Supplementary Table 4 The list of all Gene Ontology (GO) processes, which characterize HIV and HC group. GO was performed by analyses of biological processes, molecular function, cellular components as well as pathway interactions. Data was arranged from the highest statistically significant value to the lowest one.

Supplementary Table 5 The list of all tested mRNA for genes associated with activation of neutrophil *via* NF-κB. Data are presented as the relative fold differences between HIV and HC group.

Supplementary Table 6 The list of all target genes in the GO term ‘Apoptotic process’ GO:0006915, ‘Histone methyltransferase complex GO:0035097 and ‘Histone acetyltransferase complex’ GO:0000123 divided into HIV specific, HC specific and common for both groups.

## References

[1] Nathan C. Neutrophils and Immunity: Challenges and Opportunities. Nat Rev Immunol (2006) 6:173–82. 10.1038/nri1785 16498448

[2] Prodger JL Gray RH Shannon B Shahabi K Kong X Grabowski K. Chemokine Levels in the Penile Coronal Sulcus Correlate With HIV-1 Acquisition and Are Reduced by Male Circumcision in Rakai, Uganda. PloS Pathog (2016) 12(11):e1006025. 10.1371/journal.ppat.1006025 27898732 PMC5127584

[3] Hensley-McBain T Wu MC Manuzak JA Cheu RK Gustin A Driscoll CB. Increased Mucosal Neutrophil Survival Is Associated With Altered Microbiota in HIV Infection. PloS Pathog (2019) 15(4):e1007672. 10.1371/journal.ppat.1007672 30973942 PMC6459500

[4] Hunt PW. HIV and Inflammation: Mechanisms and Consequences. Curr HIV/AIDS Rep (2012) 9:139–47. 10.1007/s11904-012-0118-8 22528766

[5] Salmen S Terán G Borges L Goncalves L Albarrán B Urdaneta H. Increased Fas-Mediated Apoptosis in Polymorphonuclear Cells From HIV-Infected Patients. Clin Exp Immunol (2004) 137(1):166–72. 10.1111/j.1365-2249.2004.02503 PMC1809087 15196258

[6] Flø RW Naess A Nilsen A Harthug S Solberg CO. A Longitudinal Study of Phagocyte Function in HIV-Infected Patients. AIDS (1994) 8(6):771–7. 10.1097/00002030-199406000-00008 8086135

[7] Cloke TE Garvey L Choi BS Abebe T Hailu A Hancock M. Increased Level of Arginase Activity Correlates With Disease Severity in HIV-Seropositive Patients. J Infect Dis (2010) 202(3):374–85. 10.1086/653736 PMC4663662 20575659

[8] Bowers NL Helton ES Huijbregts RP Goepfert PA Heath SL Hel Z. Immune Suppression by Neutrophils in HIV-1 Infection: Role of PD-L1/PD-1 Pathway. PloS Pathog (2014) 10(3):e1003993. 10.1371/journal.ppat.1003993 24626392 PMC3953441

[9] Hensley-McBain T Klatt NR. The Dual Role of Neutrophils in HIV Infection. Curr HIV/AIDS Rep (2018) 15(1):1–10. 10.1007/s11904-018-0370-7 29516266 PMC6086572

[10] Campillo-Gimenez L Casulli S Dudoit Y Seang S Carcelain G Lambert-Niclot S. Neutrophils in Antiretroviral Therapy-Controlled HIV Demonstrate Hyperactivation Associated With a Specific IL-17/IL-22 Environment. J Allergy Clin Immunol (2014) 134(5):1142–52.e5. 10.1016/j.jaci.2014.05.040 25042982

[11] Natoli G Ghisletti S Barozzi I. The Genomic Landscapes of Inflammation. Genes Dev (2011) 25(2):101–6. 10.1101/gad.2018811 PMC3022255 21245163

[12] Beyaz S Kim JH Pinello L Xifaras ME Hu Y Huang J. The Histone Demethylase UTX Regulates the Lineage-Specific Epigenetic Program of Invariant Natural Killer T Cells. Nat Immunol (2017) 18:184–95. 10.1038/ni.3644 PMC5247321 27992400

[13] Bunting KL Soong TD Singh R Jiang Y Beguelin W Poloway DW. Multi-Tiered Reorganization of the Genome During B Cell Affinity Maturation Anchored by a Germinal Center-Specific Locus Control Region. Immunity (2016) 45:497–512. 10.1016/j.immuni.2016.08.012 27637145 PMC5033726

[14] Johnson JL Georgakilas G Petrovic J Kurachi M Cai S Harly C. Lineage-Determining Transcription Factor TCF-1 Initiates the Epigenetic Identity of T Cells. Immunity (2018) 48:243–57. 10.1016/j.immuni.2018.01.012 PMC5824646 29466756

[15] Northrup DL Zhao K. Application of ChIP-Seq and Related Techniques to the Study of Immune Function. Immunity (2011) 34(6):830–42. 10.1016/j.immuni.2011.06.002. 28 ed. 2003. 32: 475-481. PMC3137373 21703538

[16] Cildir G Toubia J Yip KF Zhou M Pant H Hissaria P. Genome-Wide Analyses of Chromatin State in Human Mast Cells Reveal Molecular Drivers and Mediators of Allergic and Inflammatory Diseases. Immunity (2019) 51(5):949–965.e6. 10.1016/j.immuni.2019.09.021 31653482

[17] Pennacchio LA Bickmore W Dean A Nobrega MA Bejerano G. Enhancers: Five Essential Questions. Nat Rev Genet (2013) 14(4):288–95. 10.1038/nrg3458 PMC4445073 23503198

[18] Glass C Natoli G. Molecular Control of Activation and Priming in Macrophages. Nat Immunol (2015) 17(1):26–33. 10.1038/ni.3306 PMC4795476 26681459

[19] Chen K Chen Z Wu D Zhang L Lin X Su J. Broad H3K4me3 is Associated With Increased Transcription Elongation and Enhancer Activity at Tumor-Suppressor Genes. Nat Genet (2015) 47:1149–57. 10.1038/ng.3385 PMC4780747 26301496

[20] Park S Go Woon K So Hee K Jung-Shin L. Broad Domains of Histone H3 Lysine 4 Trimethylation in Transcriptional Regulation and Disease. FEBS J (2020) 287:2891–902. 10.1111/febs.15219 31967712

[21] Lewkowicz N Lewkowicz P Kurnatowska A Banasik M Głowacka E Cedzyński M. Innate Immune System Is Implicated in Recurrent Aphthous Ulcer Pathogenesis. J Oral Pathol Med (2003) 32(8):475–81. 10.1034/j.1600-0714.2003.00181.x 12901729

[22] Piątek P Namiecinska M Domowicz M Wieczorek M Michlewska S Matysiak M. Multiple Sclerosis CD49d+CD154+ As Myelin-Specific Lymphocytes Induced During Remyelination. Cells (2020) 9(1):15. 10.3390/cells9010015 PMC7017443 31861635

[23] Egelhofer TA Minoda A Klugman S Lee K Kolasinska-Zwierz P Alekseyenko AA. An Assessment of Histone-Modification Antibody Quality. Nat Struct Mol Biol (2011) 18(1):91–3. 10.1038/nsmb.1972 PMC3017233 21131980

[24] Landt SG Marinov GK Kundaje A Kheradpour P Pauli F Batzoglou S. ChIP-Seq Guidelines and Practices of the ENCODE and modENCODE Consortia. Genome Res (2012) 22(9):1813–31. 10.1101/gr.136184.111 PMC3431496 22955991

[25] Feng JX Qin B Zhang Y Liu XS. Identifying ChIP-Seq Enrichment Using MACS. Nat Protoc (2012) 7:17. 10.1038/nprot.2012.101 PMC3868217 22936215

[26] Schaefer CF Anthony K Krupa S Buchoff J Day M Hannay T. PID: The Pathway Interaction Database. Nucleic Acids Res (2009) 37(Database issue):D674–9. 10.1093/nar/gkn653 PMC2686461 18832364

[27] Zhou VW Goren A Bernstein BE. Charting Histone Modifications and the Functional Organization of Mammalian Genomes. Nat Rev Genet (2011) 12(1):7–18. 10.1038/nrg2905 21116306

[28] Kouzarides T. Chromatin Modifications and Their Function. Cell (2007) 128(4):693–705. 10.1016/j.cell.2007.02.005 17320507

[29] Lu T Stark GR. NF-κB Regulation by Methylation. Cancer Res (2015) 75(18):3692–5. 10.1158/0008-5472.CAN-15-1022 PMC4573795 26337909

[30] Sun SC. The Non-Canonical NF-κB Pathway in Immunity and Inflammation. Nat Rev Immunol (2017) 17:545–58. 10.1038/nri.2017.52 PMC5753586 28580957

[31] Hoesel B Schmid JA. The Complexity of NF-κb Signaling in Inflammation and Cancer. Mol Cancer. (2013) 12:86. 10.1186/1476-4598-12-86 23915189 PMC3750319

[32] Pazin MJ Kadonaga JT. SWI2/SNF2 and Related Proteins: ATP-Driven Motors That Disrupt-Protein–DNA Interactions? Cell (1997) 88(6):737–40. 10.1016/S0092-8674(00)81918-2 9118215

[33] Clapier CR Iwasa J Cairns BR Peterson CL. Mechanisms of Action and Regulation of ATP-Dependent Chromatin-Remodelling Complexes. Nat Rev Mol Cell Biol (2017) 18(7):407–22. 10.1038/nrm.2017.26 PMC8127953 28512350

[34] Rothbart SB Strahl BD. Interpreting Thelanguage of Histone and DNA Modifications. Biochim Biophys Acta (2014) 1839(8):627–43. 10.1016/j.bbagrm.2014.03.001 PMC4099259 24631868

[35] Bediaga NG Coughlan HD Johanson TM Garnham AL Naselli G Schröder J. Multi-Level Remodelling of Chromatin Underlying Activation of Human T Cells. Sci Rep (2021) 11(1):528. 10.1038/s41598-020-80165-9 33436846 PMC7804404

[36] Hoffmann A Levchenko A Scott ML Baltimore D. The Iκb-NF-κb Signaling Module: Temporal Control and Selective Gene Activation. Science (2002) 298(5596):1241–5. 10.1126/science.1071914 12424381

[37] Piatek P Domowicz M Lewkowicz N Przygodzka P Matysiak M Dzitko K. C5a-Preactivated Neutrophils Are Critical for Autoimmune-Induced Astrocyte Dysregulation in Neuromyelitis Optica Spectrum Disorder. Front Immunol (2018) 9:169. 10.3389/fimmu.2018.0169 30083159 PMC6065055

[38] Tang L Nogales E Ciferrie C. Structure and Function of SWI/SNF Chromatin Remodeling Complexes and Mechanistic Implications for Transcription. Prog Biophys Mol Biol (2010) 102(2–3):122–8. 10.1016/j.pbiomolbio.2010.05.001 PMC2924208 20493208

[39] Ramirez-Carrozzi VR Nazarian AA Li CC Gore SL Sridharan R Imbalzano AN. Selective and Antagonistic Functions of SWI/SNF and Mi-2beta Nucleosome Remodeling Complexes During an Inflammatory Response. Genes Dev (2006) 20(3):282–96. 10.1101/gad.1383206 PMC1361700 16452502

[40] Liu N Balliano A Hayes JJ. Mechanism(s) of SWI/SNF-Induced Nucleosome Mobilization. Chembiochem (2011) 12(2):196–204. 10.1002/cbic.201000455 21243709 PMC3787519

[41] Ghosh S. New Regulators of NF-kappaB in Inflammation. Nat Rev Immunol (2008) 8(11):837–48. 10.1038/nri2423 18927578

[42] Gilmore TD Wolenski FS. NF-κb: Where did it Come From and Why? Immunol Rev (2012) 246(1):14–35. 10.1111/j.1600-065X.2012.01096.x 22435545

[43] Huxford T Hoffmann A Ghosh G. Understanding the Logic of Iκb:NF-κb Regulation in Structural Terms. Curr Topics Microbiol Immunol (2011) 349:1–24. 10.1007/82_2010_99 20845107

[44] Smale ST Natoli G. Transcriptional Control of Inflammatory Responses. Cold Spring Harb Perspect Biol (2014) 6(11):a016261. 10.1101/cshperspect.a016261 25213094 PMC4413233

[45] Heintzman N Stuart R Hon G Fu Y Ching C Hawkins R. Distinct and Predictive Chromatin Signatures of Transcriptional Promoters and Enhancers in the Human Genome. Nat Genet (2007) 39:311–8. 10.1038/ng1966 17277777

[46] Rada-Iglesias A Bajpai R Swigut T Brugmann SA Flynn RA Wysocka J. A Unique Chromatin Signature Uncovers Early Developmental Enhancers in Humans. Nature (2011) 470:279–83. 10.1038/nature09692 PMC4445674 21160473

[47] Elbim C Prevot M Bouscarat F Franzini E Chollet-Martin S Hakim J. Polymorphonuclear Neutrophils From Human Immunodeficiency Virus-Infected Patients Show Enhanced Activation, Diminished fMLP-Induced L -Selectin Shedding, and an Impaired Oxidative Burst After Cytokine Priming. Blood (1994) 84:2759–66. 10.1182/blood.V84.8.2759.bloodjournal8482759 7522641

[48] Bröker LE Kruyt FAE Giaccone G. Cell Death Independent of Caspases: Review. Clin Cancer Res (2005) 11(9):3155–62. 10.1158/1078-0432.CCR-04-2223 15867207

[49] Hergeth SP Schneider R. The H1 Linker Histones: Multifunctional Proteins Beyond the Nucleosomal Core Particle. EMBO Rep (2015) 16(11):1439–53. 10.15252/embr.201540749 PMC4641498 26474902

[50] Konishi A Shimizu S Hirota J Takao T Fan Y Matsuoka Y. Involvement of Histone H1.2 in Apoptosis Induced by DNA Double-Strand Breaks. Cell (2003) 114(6):673–88. 10.1016/s0092-8674(03)00719-0 14505568

[51] Giné E Crespo M Muntañola A Calpe E Baptista MJ Villamor N. Induction of Histone H1.2 Cytosolic Release in Chronic Lymphocytic Leukemia Cells After Genotoxic and Non-Genotoxic Treatment. Haematologica (2008) 93(1):75–82. 10.3324/haematol.11546 18166788

[52] Baldelli F Preziosi R Francisci D Tascini C Bistoni F Nicoletti I. Programmed Granulocyte Neutrophil Death in Patients at Different Stages of HIV Infection. AIDS (2000) 14:1067–9. 10.1097/00002030-200005260-00024 10853994

[53] Pitrak DL Tsai HC Mullane KM Sutton SH Stevens P. Accelerated Neutrophil Apoptosis in the Acquired Immunodeficiency Syndrome. J Clin Invest (1996) 98(12):2714–9. 10.1172/JCI119096 PMC507735 8981916

[54] Mastroianni CM Mengoni F Lichtner M D’Agostino C d’Ettorre G Forcina G. Ex Vivo and *In Vitro* Effect of Human Immunodeficiency Virus Protease Inhibitors on Neutrophil Apoptosis. J Infect Dis (2000) 182:1536–9. 10.1086/315858 11023478

[55] Elbim C Katsikis PD Estaquier J. Neutrophil Apoptosis During Viral Infections. Open Virol J (2009) 3:52–9. 10.2174/1874357900903010052 PMC2703832 19572056

[56] Salmen S Montes H Soyano A Hernández D Berrueta L. Mechanisms of Neutrophil Death in Human Immunodeficiency Virus-Infected Patients: Role of Reactive Oxygen Species, Caspases and Map Kinase Pathways. Clin Exp Immunol (2007) 150:539–45. 10.1111/j.1365-2249.2007.03524.x PMC2219366 17956581

[57] Casulli S Elbim C. Interactions Between Human Immunodeficiency Virus Type 1 and Polymorphonuclear Neutrophils. J Innate Immun (2014) 6(1):13–20. 10.1159/000353588 23867213 PMC6741617

[58] Billiau A Matthys P. Interferon-Gamma: A Historical Perspective. Cytokine Growth Factor Rev (2009) 20(2):97–113. 10.1016/j.cytogfr.2009.02.004 19268625

[59] Chiba Y Mizoguchi I Hasegawa H Ohashi M Orii N Nagai T. Regulation of Myelopoiesis by Proinflammatory Cytokines in Infectious Diseases. Cell Mol Life Sci (2018) 75(8):1363–76. 10.1007/s00018-017-2724-5 PMC11105622 29218601

[60] Binder D Fehr J Hengartner H Zinkernagel RM. Virus-Induced Transient Bone Marrow Aplasia: Major Role of Interferon-Alpha/Beta During Acute Infection With the Noncytopathic Lymphocytic Choriomeningitis Virus. J Exp Med (1997) 185(3):517–30. 10.1084/jem.185.3.517 PMC2196026 9053452

[61] Buro LJ Chipumuro E Henriksen MA. Menin and RNF20 Recruitment Is Associated With Dynamic Histone Modifications That Regulate Signal Transducer and Activator of Transcription 1 (STAT1)-Activated Transcription of the Interferon Regulatory Factor 1 Gene (IRF1). Epigenet Chromatin (2010) 3(1):16. 10.1186/1756-8935-3-16 PMC2940767 20825659

[62] Essers MA Offner S Blanco-Bose WE Waibler Z Kalinke U Duchosal MA. IFNalpha Activates Dormant Haematopoietic Stem Cells *In Vivo* . Nature (2009) 458(7240):904–8. 10.1038/nature07815 19212321

[63] Lu T Yang M Huang DB Wei H Ozer GH Ghosh G. Role of Lysine Methylation of NF-κb in Differential Gene Regulation. Proc Natl Acad Sci USA (2013) 110(33):13510–5. 10.1073/pnas.1311770110 PMC3746872 23904479

