# Supplementary figures and images for: H3K4me3 Histone ChIP-Seq Analysis Reveals Molecular Mechanisms Responsible for Neutrophil Dysfunction in HIV-Infected Individuals
Source: Front Immunol. 2021 Jul 15;12:682094. doi: 10.3389/fimmu.2021.682094 (PMC8320512; doi:10.3389/fimmu.2021.682094)

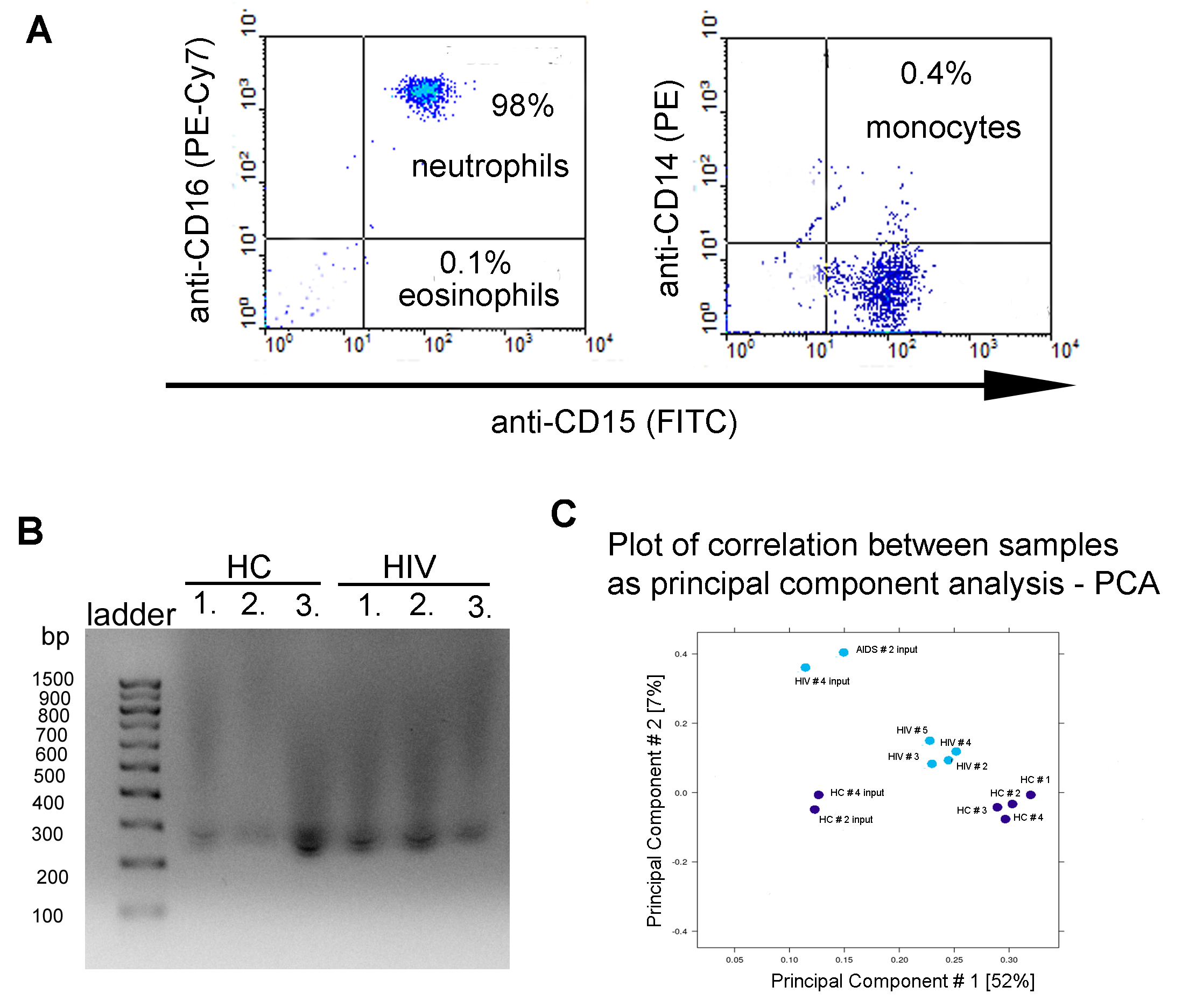

Supplement: Supplementary Figure 1 — (A). Phenotypic analysis of purified neutrophils used for ChIPseq experiments. Flow cytometry analysis of CD14, CD15 and CD16 in the population of isolated neutrophils. Cells double-positive for CD16 and CD15 were identified as neutrophils, CD14highCD15+ cells as monocytes and with CD16-CD15+ cells as eosinophils. (B). ChIPseq library quality control analysis. ChIPseq library was generated from 1 ng ChIP DNA with 18 cycles of PCR. The size of the library distribution is approximately 200-300 bp. (C). ChIPseq quality control report as principal component analysis (PCA). Replicate samples of high quality can be expected to be spatially grouped within the PCA plot. Evaluation of the PCA plot has shown that all samples are suitable and representative for bioinformatic analysis. The analysis was performed by ChIPQC:1.21.0 software (author: Tom Carroll, Wei Liu, Ines de Santiago, Rory Stark). [file Image_1.tif]
