# Supplementary material for: H3K4me3 Histone ChIP-Seq Analysis Reveals Molecular Mechanisms Responsible for Neutrophil Dysfunction in HIV-Infected Individuals
Source: Front Immunol. 2021 Jul 15;12:682094. doi: 10.3389/fimmu.2021.682094 (PMC8320512; doi:10.3389/fimmu.2021.682094)
Supplement: Supplementary Table 1 — The profile of cytokines/chemokines/growth factors released by non- and LPS-stimulated neutrophils in healthy controls and HIV-infected individuals. Data are presented in pg/mL as means ± SD. [file Table_1.doc]

**Table S1.** The profile of cytokines/chemokines/growth factors released by non- and LPS- stimulated neutrophils in healthy controls (HC) and HIV-infected individuals. Data are presented in pg/mL as means ± SD.

|  | Neutrophils (2x10^6^ cells/mL) | | | |
| --- | --- | --- | --- | --- |
|  | HC (n=12) | | HIV (n=24) | |
|  | n.s. | LPS | n.s. | LPS |
| **IL-1β** | n.d. | 27.7 ± 27.41 | n.d. | 7.9 ± 9.33 |
| **IL-1ra** | 354.1 ± 69.96 | 2336.4 ± 878.79 | 184.6 ± 57.35 † | 1262.5 ± 640.37 † |
| **IL-2** | n.d. | 42.2 ± 22.89 | n.d. | 22.5 ± 10.01 † |
| **IL-4** | n.d. | 15.6 ± 9.25 | n.d. | 5.1 ± 4.91 † |
| **IL-5** | 7.6 ± 0 .95 | 168.7 ± 65.25 | 5.7 ± 1.94 | 88.7 ± 47.49 † |
| **IL-6** | n.d. | 297.6 ± 207.51 | n.d. | 200.2 ± 237.71 |
| **IL-7** | n.d. | 8.2 ± 4.62 | n.d. | 1.6 ± 1.71 † |
| **IL-8** | 31.4 ± 10.62 | 5906.9 ± 3352.93 | 78.0 ± 55.94 † | 2473.9 ± 1930.48 † |
| **IL-9** | 2.8 ± 1.45 | 55.4 ± 21.74 | 7.6 ± 6.73 | 25.1 ± 17.06 † |
| **IL-10** | n.d. | 40.2 ± 13.81 | n.d. | 20.2 ± 12.77 † |
| **IL-12** | n.d. | 6.9 ± 7.59 | n.d. | n.d. |
| **IL-13** | n.d. | n.d. | n.d. | n.d. |
| **IL-15** | 54.1 ± 6.13 | 1105.4 ± 378.10 | 47.9 ± 11.73 | 664.7 ± 322.42 † |
| **IL-17** | n.d. | 187.2 ± 68.33 | n.d. | 51.8 ± 48.94 † |
| **Eotaxin** | n.d. | 81.2 ± 57.92 | n.d. | 23.1 ± 20.31 † |
| **FGF basic** | n.d. | 355.2 ± 153.73 | n.d. | 151.5 ± 127.64 † |
| **G-CSF** | 66.4 ± 46.15 | 14047.6 ± 6336.21 | 120.1 ±107.02 † | 6271.4 ± 5518.63 † |
| **GM-CSF** | n.d. | 16.9 ± 7.16 | n.d. | 5.2 ± 4.70 † |
| **IFN-γ** | 11.9 ± 2.33 | 146.0 ± 53.90 | 19.4 ± 2.23 † | 293.0 ± 34.38 † |
| **IP-10** | n.d. | 76.7± 40.42 | n.d. | 19.8 ± 28.23 † |
| **MCP-1** | n.d. | 51.9 ± 22.48 | n.d. | 20.6 ± 18.98 † |
| **MIP-1α** | 1.7 ± 1.01 | 743.4 ± 613.01 | n.d. | 251.0 ± 187.73 † |
| **MIP-1β** | 10.2 ± 11.36 | 2550.4 ± 1840.19 | 8.2 ± 10.87 | 717.4 ± 685.41 † |
| **PDGF-bb** | n.d. | 267.7 ±141.21 | n.d. | 81.5 ± 79.86 † |
| **RANTES** | 6.5 ± 3.72 | 41.8 ± 15.06 | 8.6 ± 5.99 | 27.6 ± 12.56 † |
| **TNF** | 2.4 ± 1.22 | 497.7 ± 258.64 | 2.5 ± 3.21 | 299.3 ± 179.85 † |
| **VEGF** | 75.6 ± 14.46 | 1740.4 ± 601.19 | 70.6 ± 19.02 | 1056.6 ± 555.49 † |

† - statistically significant differences to HC group

red font - chemokines (associated with recruitment of neutrophils and lymphocytes)

blue font – pro-inflammatory cytokines

green font – anti-inflammatory mediators
